# Thoracic limb myology of *Smutsia temminckii* (Temminck's pangolin)

**DOI:** 10.1111/joa.70268

**Published:** 2026-11-14

**Authors:** Christine Steyn, John Thomson Soley, Martina Rachel Crole

**Affiliations:** ^1^ Department of Anatomy and Physiology, Faculty of Veterinary Science University of Pretoria Onderstepoort South Africa

**Keywords:** *M. omoradialis*, muscle maps, Pholidota, scratch digging, thoracic limb

## Abstract

The myrmecophagous *Smutsia temminckii* (Temminck's pangolin) is one of eight pangolin species, of threatened status worldwide and the only species present in southern Africa. *S. temminckii* utilises a bipedal gait for locomotion and will roll into a ball when vulnerable. The thoracic limbs are predominantly used to assist in exposing prey by employing scratch‐digging in superficial substrates and breaking open ant nests. This study describes the thoracic limb musculature and muscle maps in *S. temminckii*, as well as discusses the peculiar adaptations of the thoracic limb to its unique lifestyle. The thoracic limbs of five *S. temminckii* were donated after they succumbed to either natural causes, electrocution or confiscation from the illegal wildlife trade. All ethical clearances were received. The muscles of the thoracic limbs were dissected and described, after which the bones were cleaned and the areas of muscle attachment identified and described. The muscles were generally well‐developed with fleshy bodies and short tendons. The extrinsic muscles of the thoracic limb, and in particular *M. cutaneous trunci*, were prominent. *M. triceps brachii*, *M. pronator teres* and *M. flexor digitorum profundus* displayed additional bellies and extensive attachments. Individual metacarpophalangeal and digital muscles were noted. Two muscles were specifically named: the fleshy *M. anconeus medialis* and the long *M. omoradialis*. The musculature of *S. temminckii* shows clear adaptations to protraction and retraction of the forelimb, extension of the elbow joint, pronation and supination of the antebrachium, and flexion of the carpus and digits, which is consistent with scratch‐digging, instead of weight‐bearing. Both protractors of the forelimb and the prominent cutaneous musculature are involved when *S. temminckii* rolls up into a sphere during its defence posture. The muscle maps generated during this study aid in understanding the various muscle functions, as well as providing valuable information for postulating muscles in extinct pangolin species. The study may also aid workers in the veterinary field when treating musculoskeletal conditions in injured pangolins.

## Graphical abstract

The thoracic limb musculature of *Smutsia temminckii* is specialised for defensive rolling, powerful limb retraction, elbow extension, digital flexion and antebrachial rotation, supporting its adaptation as a scratch‐digging mammal. These findings provide a valuable anatomical basis for veterinary care and future comparative studies of pangolins.

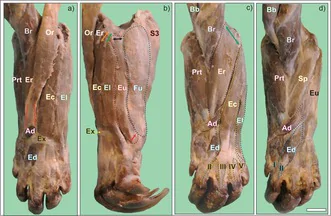

## 1 INTRODUCTION

*Smutsia temminckii* (Temminck's pangolin) is one of eight pangolin species and the only one present in South Africa (Bräutigam et al., 1994; Swart, 2013). It is a myrmecophagous mammal (Swart, 2013; Sweeney, 1956) and uses its thoracic limbs to dig for prey species in shallow soil (Richer et al., 1997; Swart, 2013), break open ant and termite nests (Hoyt, 1987; Robinson, 2003) and expand previously occupied burrows (Heath, 1992b; Kingdon, 1971; Richer et al., 1997). *S*. *temminckii* moves with a bipedal gait (Kingdon, 1971; Swart, 2013) and can roll up into a ball when threatened, thus exposing its hard, overlapping scales to the enemy (Heath, 1992a, 1992b).

Ant‐eating mammals include pangolins (Pholidota), as well as the ground‐dwelling aardvark (Tubulidentata), anteaters (Vermilingua) and armadillos (Cingulata) (Delsuc et al., 2014). Pholidota species are either arboreal, such as *Phataginus tricuspis* (white‐bellied pangolin) and *Phataginus tetradactyla* (long‐tailed pangolin), or ground‐dwelling, such as *S. temminckii* and *Mantis pentadactyla* (Chinese pangolin) (Pangolin Specialist Group, 2016; Swart, 2013). Although most myrmecophagous mammals use their thoracic limbs for both locomotion and digging, *S. temminckii* uses its thoracic limbs solely for digging (Heath, 1992b; Swart, 2013). This singularity could be related to some anatomical peculiarities associated with its locomotion, particularly given that its body weight is not supported by its thoracic limbs. Mammals that feed on ants have developed similar adaptations such as elongated, sticky tongues as well as powerful claws (Delsuc et al., 2014) and have thus prompted numerous morphological comparisons of the locomotory system between these diverse orders (Humphry, 1869; Kawashima et al., 2015; Marshall et al., 2021; Shrivastava, 1962; Windle & Parsons, 1899). Pholidota species, such as *P. tricuspis*, *P. tetradactyla*, *M. pentadactyla* and *Manis javanica* (Malayan pangolin), have been compared with other unrelated myrmecophagous mammals such as *Orycteropus afer* (aardvark) (Shrivastava, 1962; Windle & Parsons, 1899), *Myrmecophaga tridactyla* (giant anteater) (Windle & Parsons, 1899), *Tamandua tetradactyla* (lesser anteater) (Shrivastava, 1962; Windle & Parsons, 1899), *Cyclopes didactylus* (silky anteater) (Humphry, 1869; Shrivastava, 1962; Windle & Parsons, 1899) and a variety of Cingulata species (Kawashima et al., 2015; Marshall et al., 2021; Shrivastava, 1962; Windle & Parsons, 1899). *S*. *temminckii* was not included in the above‐mentioned studies and is the only pangolin species to display a bipedal gait, making it unique amongst the Pholidota.

The ant‐eating mammals mentioned above, including *S*. *temminckii*, use their powerful claws to break open ant nests and dig in the soil for ants and termites (Delsuc et al., 2014). Specific muscular adaptations in digging mammals include muscles that attach relatively far from the joints on which they act, as well as extensive muscle origins and insertions (Hildebrand & Goslow, 2001b). Furthermore, different forms of digging have been identified, namely scratch‐digging (Tubulidentata, Cingulata, Pholidota and *Bathyergus* species [dune mole rats]), hook‐and‐pull digging (Vermilingua), as well as chisel‐tooth digging (*Geomyidae* [gophers] and *Bathyergidae* [African mole‐rats]), and humeral‐rotation digging (*Talpidae* [true moles]) (Hildebrand & Goslow, 2001b). Muscular adaptations that are specific to scratch‐diggers include well‐developed thoracic limb retractors, elbow extensors and carpal and digital flexors (Endo et al., 2002; Marshall et al., 2021; Olson et al., 2016; Thewissen & Badoux, 1986; Warburton et al., 2013). Based on the observation that the bones of the thoracic limb of *S. temminckii* are adapted to support scratch‐digging (Steyn et al., 2018), corresponding muscular adaptations would accordingly be expected in this species.

Morphological muscular adaptations of the thoracic limb to scratch‐digging have been described in *O*. *afer* (Thewissen & Badoux, 1986), *Dasypus novemcinctus* (nine‐banded armadillo) (Olson et al., 2016), and other Cingulata species (Marshall et al., 2021). Thoracic limb myology of certain sloth species, such as *Choloepus didactylus* (Linnaeus's two‐toed sloth) and *Bradypus tridactylus* (pale‐throated three‐toed sloth), has previously been compared with that of more typical myrmecophagous mammals, including Vermilingua, with which they are grouped in the same order, the Pilosa (Humphry, 1869; Shrivastava, 1962; Windle & Parsons, 1899). The functional adaptability of the thoracic limb of the sloth has also been examined (Mendel, 1981; Miller, 1935; Nyakatura, 2012; Nyakatura & Fischer, 2011; Olson et al., 2018; Tucker, 2023), and it has been demonstrated that its thoracic limb myology is related to the inverse orientation of the body with regards to the force of gravity (Nyakatura, 2012; Nyakatura & Fischer, 2011). These animals are therefore adapted to an arboreal lifestyle in their mode of locomotion (Miller, 1935), and despite sloths possessing large claws similar to myrmecophagous mammals, they may not be a fitting comparison to Pholidota, as some previous studies have attempted (Humphry, 1869; Shrivastava, 1962; Windle & Parsons, 1899). Muscular adaptations of the thoracic limb to scratch digging have also been described in rodents such as *Bathyergus suillus* (Cape dune mole‐rat) (Doubell et al., 2020; Montoya‐Sanhueza et al., 2024), *Bathyergus janetta* (Namaqualand dune mole‐rat) (Montoya‐Sanhueza et al., 2024), *Spermophilus citellus* (European ground squirrel) (Lagaria & Youlatos, 2006) and *Cynomys ludovicianus* (black‐tailed prairie dog) (Peterka, 1936; Scheetz et al., 2026), in marsupials such as *Isoodon obesulus* (southern brown bandicoot) and *Macrotis lagotis* (greater bilby) (Warburton et al., 2013) and in carnivores such as *Taxidea taxus* (American badger) (Moore, 2011; Moore et al., 2013). The functional adaptability of the above‐mentioned species may provide more useful comparisons to specific features noted in *S*. *temminckii*.

In a broader context, muscular specialisations and adaptations of the thoracic limb have been recently described in numerous mammalian species (de Souza Junior et al., 2021; Kabkia et al., 2022; Marshall et al., 2021; Rose et al., 2013; Williams et al., 2007), including various carnivores such as *Acinonyx jubatus* (cheetah) (Hudson et al., 2011), *Ailurus fulgens* (red panda) (Fisher et al., 2009), *Canis familiaris* (domestic dog) (Williams et al., 2008), *Galictis cuja* (lesser grison) (Ercoli et al., 2015), *Leopardus geoffroyi* (Geoffroy's cat) (Cardozo et al., 2026; Sánchez et al., 2019), *Leopardus pardalis* (ocelot) (Julik et al., 2012; Sánchez et al., 2019), *Leopardus wiedii* (margay cat) (da da Costa da Silva et al., 2025), *Lycalopex gymnocercus* (pampas fox) (de Souza Junior et al., 2018), *Nasua nasua* (ring‐tailed coati) (Tarquini et al., 2023), *Panthera leo* (lion) (Vargas et al., 2017), *Panthera onca* (jaguar) (Sánchez et al., 2019), *Panthera tigris* (tiger) (Dunn et al., 2022) and *Panthera uncia* (snow leopard) (Smith et al., 2021). Mapping of muscles onto the bones of the thoracic limb provides insight into the functional adaptations of the various species (de Souza Junior et al., 2018; Dunn et al., 2022; Hudson et al., 2011; Marshall et al., 2021; Smith et al., 2021). Based on the osteology of the thoracic limb in *S. temminckii* (Steyn et al., 2018), this study will use a similar technique to elucidate the functional adaptations of thoracic limb myology in this species.

Historically, it was believed that the Edentata order (no incisors and no/poorly developed molars) included Pholidota, Tubulidentata and Xenarthra (armadillos, tree sloths and anteaters) (Humphry, 1869; Shoshani, 1986; Shoshani & McKenna, 1998; Smith, 1899). However, recent genetic studies have determined that the similarities between the species result from convergent evolution (Reiss, 2001). Pangolins are now grouped in a separate order and are placed in a monophyletic grouping with the Carnivora order to form the superordinal clade Ferae (Amrine‐Madson et al., 2003; Arnason et al., 2002; Du Toit et al., 2014; Perelman et al., 2012; Reiss, 2001; Shoshani & McKenna, 1998).

No comprehensive information is available on the thoracic limb myology of *S*. *temminckii*. The aim of this study is to provide a detailed gross morphological description of the extrinsic muscles and thoracic limb musculature of *S*. *temminckii*. It is expected that the thoracic limb myology will display adaptations to scratch‐digging and will share anatomical features similar to those of other scratch‐digging mammals, even though Pholidota are phylogenetically more closely related to Carnivora. Additionally, the thoracic limb myology may demonstrate adaptations for non‐weight‐bearing as well as adaptations that allow *S*. *temminckii* to adopt a defensive posture through ventral flexion to protect vital organs. *S*. *temminckii* is an endangered species (CITES, 2025; IUCN Red List, 2025; Pietersen et al., 2014), and a thorough knowledge of the normal anatomy will be critical for the adequate treatment and rehabilitation of these animals.

## 2 MATERIALS AND METHODS

Five *S*. *temminckii*, of either sex and varying weight (3.0–8.3 kg), were obtained from Tswalu Kalahari Game Reserve (Kalahari, Kuruman, Northern Cape Province, South Africa), or were confiscated by the South African Police Service (SAPS) from the illicit trade. The *S*. *temminckii* from Tswalu succumbed to either electrocution or natural causes, and those from the illicit trade were transported to FreeMe Wildlife Rehabilitation Non‐Profit Company (NPC 2004/000431/08) (Johannesburg, South Africa), where, despite treatment, they died from starvation and dehydration. The animals were frozen on site and transported (Transport Permit No: FAUNA 714/2012) by a member of the African Pangolin Working Group (APWG), a registered Non‐Profit Organisation (NPO 123–147) (Pretoria, South Africa). The bodies were initially stored at the National Zoological Gardens of South Africa (NZG) Biobank in Pretoria, South Africa. They were collected from the Biobank and transported to the freezer facilities of the Department of Anatomy and Physiology, Faculty of Veterinary Science, University of Pretoria (Pretoria, South Africa) for the removal of the limbs (UP Registration Certificate 07137 & Standing permit 03007). The study was approved by the Animal Ethics Committee of the University of Pretoria (V081‐14).

The frozen cadavers were partially thawed in a fridge for 72 h in the Department of Anatomy and Physiology. The skin, together with the scales, was removed from the lateral and dorsal surfaces of the head and all surfaces of the neck, trunk, thoracic limbs and abdomen, up to the mid‐lumbar region. The fat and fascia over the skinned area were removed and the cutaneous, extrinsic and pectoral girdle muscles identified. The origins and insertions were identified and described, and the relative size and shape of the muscles were described and photographed. The extrinsic muscles were then transected to facilitate removal of the thoracic limbs from the trunk. The freed thoracic limbs (*n* = 10) were subsequently immersion‐fixed in 10% neutral‐buffered formalin. The bodies were refrozen and returned to the NZG Biobank for storage.

The limbs were rinsed in running tap water for at least 48 h prior to each dissection, and all specimens were used to provide information on the musculature of the thoracic limb. This was achieved by identifying the different muscles and grouping them according to functional muscle groups, defined as the extrinsic muscles of the thoracic limb, muscles of the shoulder joint, elbow joint, radio‐ulnar joints and forepaw, respectively. The origins, insertions and extent of the different muscles were exposed, and the relative size and shape of the muscles were noted before they were described and photographed. The muscles were sequentially dissected as above and, where appropriate, removed to expose the deeper‐lying muscles. Origins, insertions and functions of the muscles have been consolidated in tabular form and included as Supporting Information.

All visual information was digitally recorded using either a Nikon1 digital camera (Nikon, Tokyo, Japan) with a 6.7–13 mm Nikon lens (Nikon, Tokyo, Japan) or a Canon 400D DSLR digital camera (Canon, Tokyo, Japan) with an 18–200 mm f/3.5–6.3 Tamron lens (Tamron, Saitama, Japan).

After removal of the muscles, the limbs were secured in a muslin cloth bag and boiled for 48 h in a stainless‐steel catering pot to remove any remaining soft tissue and expose the individual bones. The various bones of the thoracic limb were then studied, and the size, shape and sites of origin and insertion of the different muscles were noted and described (see Steyn et al., 2018).

The terminology used in this study follows that of *Nomina Anatomica Veterinaria* (N. A. V.), 6th Edition (Revised) (ICVGAN, 2017), and was selected because veterinarians in the clinical setting are more familiar with these terms. Muscles not listed in N. A. V. were named according to standardised naming conventions. Differences in terminology from other studies will be discussed. The terms ‘poorly/moderately/well‐developed’ have been used to describe the relative size of muscles, where appropriate, based on visual comparison of the various forelimb muscles.

## 3 RESULTS

A summary of all the muscles described below is found in the Supporting Information (Tables S1). Muscles identified in *S*. *temminckii* but not listed in N. A. V. are indicated with an asterisk (*) in the text, Table 1, and S1. A summary of the species compared in the study is also provided (Table S7).

**TABLE 1:** List of alternative names for front limb musculature in various relevant species presented in previous studies compared to those catalogued in N.A.V., as well as muscles (*) identified in *S. temminckii* not named in N.A.V.

N. A. V. term for muscles	Alternative terms for muscles
Extrinsic muscles of the thoracic limb	
*M. trapezius pars cervicalis*	*M. cephalohumeralis* ^4^
*M. omocephalicus* ^a^	*M. occipitoscapularis* ^2,9^
*M. omomastoideus* ^a^	*M. mastoscapularis* ^*2*,9^
*M. omotransversarius* (not present in *S. temminckii*)	*M. acromiobasilaris* ^1^ *M. cervicohumeralis* ^2^ *M. levator scapulae minor* ^4^ *M. rhomboideus profundus* ^4^
*M. rhomboideus capitis*	*M. rhomboideus minor* ^1^
*M. rhomboideus cervicis*	*M. rhomboideus major* ^1^ *M. rhomboideus colli* ^4^
*M. serratus ventralis cervicis*	*M. levator anguli scapulae* ^1^ *M. levator scapulae* ^2,5,9^ *M. serratus ventralis colli* ^4^
*M. serratus ventralis thoracis*	*M. serratus magnus* ^1,2,4^ *M. serratus anterior* ^9^
*M. brachiocephalicus*	*M. cephalohumeralis* ^4^
*M. cutaneus trunci*	*M. brachiolateralis* ^2,9^ *M. abdominohumeralis* ^4^ *M. panniculus carnosus* ^5^
*Mm. pectorales superficiales*	*M. pectoralis major* ^1,2,9^
*M. pectoralis profundus*	*M. pectoralis minor* ^1,2,9^
*M. rectus sternalis* ^a^ ^,^ ^b^	
Muscles of the shoulder joint	
*M. omoradialis* ^a^ (The muscle also acts on the elbow and radio‐ulnar joints)	*M. supinator longus* ^2,4^ (As described in other pangolin species)
Muscles of the elbow joint	
*M. tensor fasciae antebrachii*	*M. dorso‐epitrochlien* ^1^ *M. latissimo‐olecranalis* ^4^ *M. doris epitrochlearis* ^5^
*M. anconeus*	*M. anconeus externus* ^2,9^
*M. anconeus medialis* ^a^	*M. epitrochleo‐anconeus* ^1,3^ *M. anconeus internus* ^2,8^ *M. epitrochleo‐olecranalis* ^4^ *M. anconeus epitrochlearis* ^6,7^ *M. triceps brachii caput mediale accessorium* ^8,11^
*M. brachialis*	*M. brachialis anticus* ^1,2,4^
Muscles of the radio‐ulnar joints	
*M. supinator*	*M. supinator brevis* ^1,2,4^
Muscles of the carpal and digital joints	
*M. brachioradialis* (not present in *S. temminckii*)	*M. supinator longus* ^1,2,4,5,9^ (Described in species other than pangolins)
*M. extensor digitorum lateralis*	*M. extensor annularis* ^1^ *M. extensor digiti minimi* ^8^ *M. abductor digiti V longus* ^10^
*M. extensor digiti I et II*	*M. extensor indicis* ^2^
*M. abductor digiti I longus*	*M. extensor ossis metacarpi pollicis* ^1,4^ *M. extensor pollicis primus* ^2^
*M. flexor digitorum superficialis*	*M. flexor digitorum sublimis* ^1,2 4,5^
*M. abductor digiti V*	*M. abductor minimi digiti* ^2^
*M. flexor digiti V*	*M. flexor brevis minimi digiti* ^2^

*Note*: None of the earlier studies referred to in this paper describes the thoracic limb myology of *S. temminckii*. Muscles that have universally accepted names, e.g. *M. supraspinatus*, *M. biceps brachii* and *M. flexor digitorum profundus*, are not listed in the table. ^1^Galton (1869), ^2^Humphry (1869), ^3^Galton (1874), ^4^Windle and Parsons (1899), ^5^Peterka (1936), ^6^Dellon (1986), ^7^O'Driscoll et al. (1991), ^8^Ercoli et al. (2015), ^9^Kawashima et al. (2015), ^10^Olson et al. (2016), ^11^Marshall et al. (2021). Additional references, where appropriate, are given in the text.

^a^ Muscles that have been identified in *S. temminckii* but are not described in N.A.V. Each muscle is named according to its origin, insertion and/or position.

^b^ This muscle has been named according to the highly variable and inconsistent *M. rectus sternalis* present in humans. It is of academic interest only. For a detailed explanation of the *M. rectus sternalis* in humans, refer to Vishal et al. (2013) and Bilodi et al. (2015).

### 3.1 Extrinsic muscles of the thoracic limb

The extrinsic muscles of the thoracic limb secure the limbs to the trunk and include some cutaneous and pectoral girdle muscles. The sequence of the muscles described is based on their attachment and placement around the *scapula* or the *humerus*, respectively. All the muscles were prominent and well‐developed. The main functions of these muscles, besides securing the limb to the trunk, are either rotation of the *scapula* or, by their action on the *humerus*, protracting, retracting or adducting the limb.

#### 3.1.1 *M. trapezius* (Figures 1a and 2a,b) (Tables 1 and S1)

**FIGURE 1:**
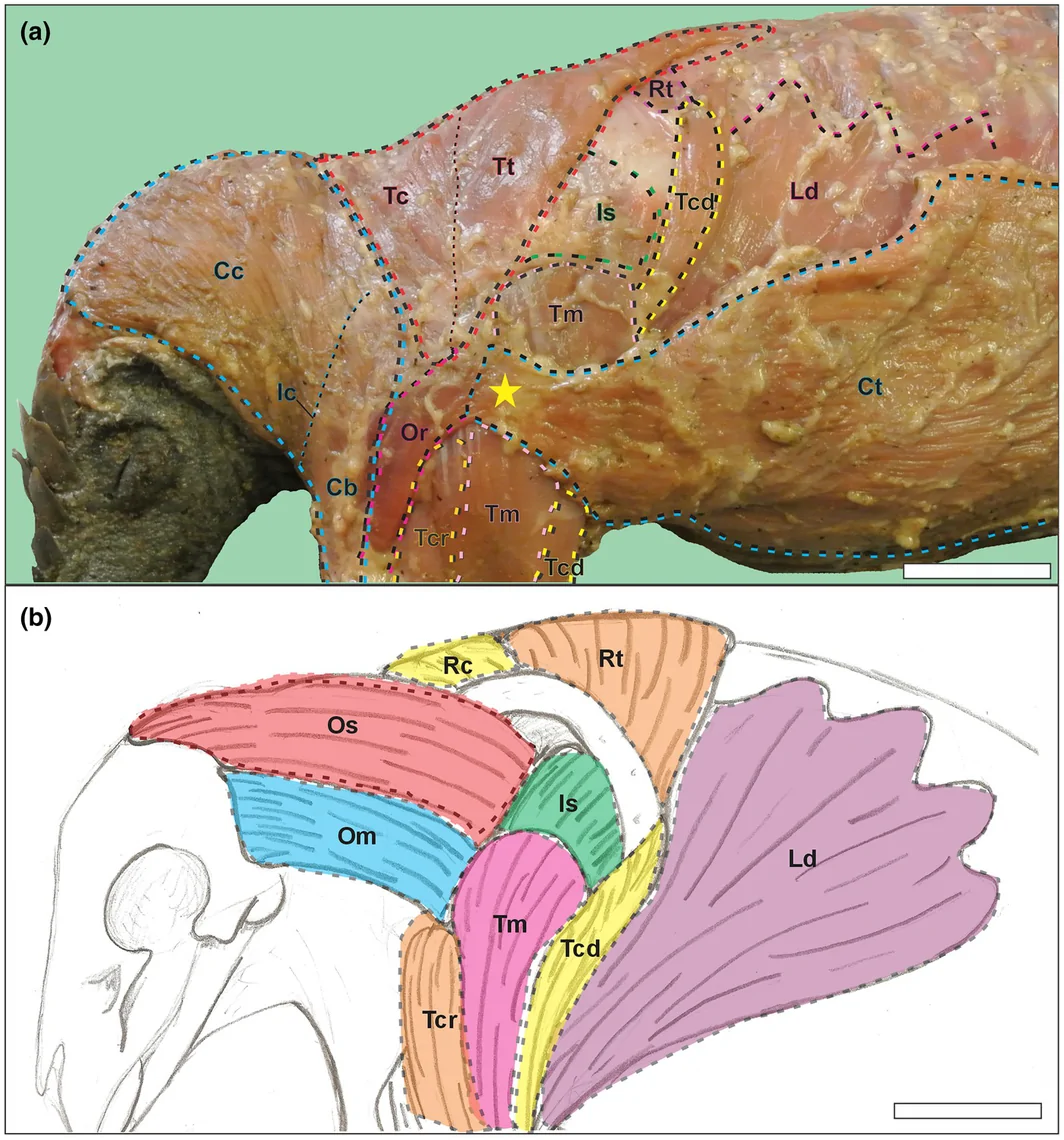
Left lateral view of the extrinsic and lateral shoulder muscles of a 7.3 kg Temminck's pangolin; (a) superficial view; (b) (schematic) deep view. (a) *Intersectio clavicularis* (Ic), *M. cleidobrachialis* (Cb), *M. cleidocephalicus* (Cc), *M. cutaneus trunci* (Ct), *M. infraspinatus* (Is), *M. latissimus dorsi* (Ld), *M. omoradialis* (Or), *M. rhomboideus thoracis* (Rt), *M. trapezius Pars cervicalis* (Tc), *M. trapezius Pars thoracica* (Tt), *M. triceps brachii Caput longum Venter caudalis* (Tcd), *M. triceps brachii Caput longum Venter cranialis* (Tcr) and *M. triceps brachii Caput longum Venter medius* (Tm). The yellow star indicates the part of the *M. cutaneus trunci* that inserts on the caudal margin of the *M. omoradialis*. (b) *M. omocephalicus Pars superficialis* (Os), *M. omomastoideus* (Om), *M. rhomboideus cervicis* (Rc) and *M. rhomboideus thoracis* (Rt). Remaining annotations as for (a). Bar = 2 cm.

**FIGURE 2:**
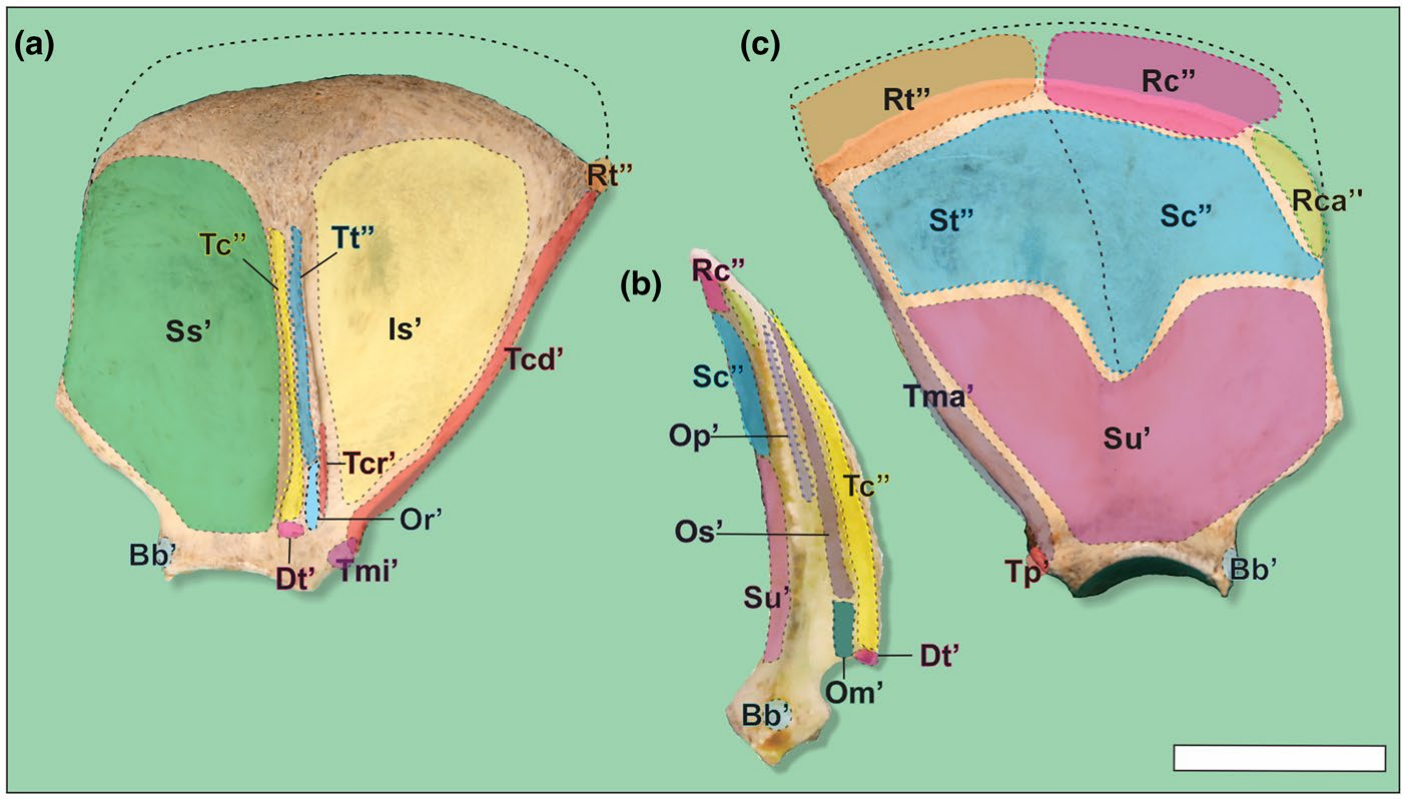
Sites of muscle origin (′) and insertion (″) on the left *Scapula* of an 8.3 kg Temminck's pangolin; (a) *Facies lateralis*; (b) Cranial view; (c) *Facies costalis*. (a) *M. biceps brachii* (Bb), *M. deltoideus* (Dt), *M. infraspinatus* (Is), *M. omoradialis* (Or), *M. rhomboideus thoracis* (Rt), *M. supraspinatus* (Ss), *M. teres minor* (Tmi), *M. trapezius Pars cervicalis* (Tc), *M. trapezius Pars thoracica* (Tt), *M. triceps brachii Caput longum Venter caudalis* (Tcd) and *M. triceps brachii Caput longum Venter cranialis* (Tcr). The dotted line dorsally represents the outline of the *Cartilago scapulae*. (b) *M. omocephalicus Pars profunda* (Op), *M. omocephalicus Pars superficialis* (Os), *M. omomastoideus* (Om), *M. rhomboideus cervicis* (Rc), *M. serratus ventralis cervicis* (Sc) and *M. subscapularis* (Su). Remaining annotations are as follows: (a). (c) *M. teres major* (Tma), *M. rhomboideus capitis* (Rca), *M. rhomboideus thoracis* (Rt), *M. serratus ventralis thoracis* (St) and *M. triceps brachii Caput longum Venter profundus* (Tp). Remaining annotations as for (a and b). Bar = 2 cm.

*M. trapezius* is a flat, triangular muscle that lies dorso‐laterally and superficial in the neck, interscapular and thoracic region. It is composed of a *Pars cervicalis* and *Pars thoracica* (Figure 1a). *Pars cervicalis* originates from the thin median raphe of the neck at the level of vertebrae C3 to C7 and from the origin of the contralateral muscle in the dorsal midline. *Pars thoracica* originates from the *Lig. supraspinale* at the level of vertebrae T1 to T10, from the cranial aponeurosis of *M. serratus dorsalis caudalis* as well as from the contralateral muscle in the dorsal midline. *Pars cervicalis* and *Pars thoracica* demonstrate different fibre directions; *Pars cervicalis* fibres run in a caudo‐ventral direction, and the fibres of the larger *Pars thoracica* run cranio‐ventrally. The cranial margin of *Pars cervicalis* is fused to the caudal border of *M. cleidocephalicus* in the lateral neck region (see Section 3.1.6). *Pars cervicalis* inserts along the length of the cranial margin of the *Spina scapulae* as well as on the cranial surface of the acromion (Figure 2a,b), superficial to the origins of *M. omocephalicus* (see Section 3.1.2) and *M. omomastoideus* (see Section 3.1.3). *Pars thoracica* inserts along the caudal surface of the scapular spine (Figure 2a). *Pars cervicalis* rotates the *Angulus ventralis* of the *Scapula* cranially, causing protraction of the limb, whereas *Pars thoracica* will draw the ventral angle caudally, thereby retracting the limb. Both parts contracting together will lift the *Scapula* dorsally and raise the thoracic limbs.

#### 3.1.2 M. omocephalicus* (Figures 1b and 2b) (Tables 1 and S1) (alternative name: *M. occipitoscapularis*)

This triangular muscle, named here *M. omocephalicus* (see Discussion and Table 1), lies deep to *M. trapezius Pars cervicalis* in the dorso‐lateral neck region (Figure 1b) and covers *M. rhomboideus capitis* in the lateral neck region (see Section 3.1.4). This muscle displays two parts. The larger *Pars superficialis* originates along the length of the cranial margin of the scapular spine, and the smaller *Pars profunda* originates on the dorsal third of the cranial margin of the scapular spine (Figure 2b). The muscle fibres run cranio‐dorsally. *Pars superficialis* displays a broad insertion dorso‐medially along half the length of the *Crista nuchae* of the *Os occipitale*, superficial to the insertion of *M. rhomboideus capitis*. *Pars profunda* inserts centrally on the *Crista nuchae*, proximal to the insertion of *M. omomastoideus* (see Section 3.1.3). Both parts are covered by the insertion of *M. cleidocephalicus* (see Section 3.1.6). This muscle will draw the ventral angle of the *Scapula* cranially, thereby protracting the limb.

#### 3.1.3 M. omomastoideus* (Figures 1b and 2b) (Tables 1 and S1) (alternative name: *M. mastoscapularis*)

This strap‐like muscle, named here *M. omomastoideus* (see Discussion and Table 1), lies dorso‐laterally in the neck region, deep to *M. cleidocephalicus* and ventral to *M. omocephalicus* (Figure 1b). The muscle originates on the cranio‐ventral part of the scapular spine (Figure 2b), distal to the origin of *M. omocephalicus* and medial to the insertion of *M. trapezius Pars cervicalis*. The muscle fibres run cranio‐dorsally. Insertion is ventro‐laterally on the *Crista nuchae* as well as on the *Os temporale Processus mastoideus*. The muscle protracts the limb by rotating the ventral angle of the *Scapula* cranially.

#### 3.1.4 *M. rhomboideus* (Figures 1a,b and 2a–c) (Tables 1 and S1)

This muscle is composed of *M. rhomboideus capitis* (Alternative name: *M. rhomboideus minor*), *M. rhomboideus cervicis* (Alternative names: *M. rhomboideus major/colli*), and *M. rhomboideus thoracis*. *M. rhomboideus capitis* is covered by, and partially fused to, *M. omocephalicus Pars superficialis* in the lateral neck region, whereas *M. rhomboideus cervicis* and *M. rhomboideus thoracis* lie deep to *M. trapezius Pars cervicalis* and *Pars thoracica* in the dorso‐lateral neck and interscapular region, respectively (Figure 1a,b). The well‐developed *M. rhomboideus capitis* originates dorso‐medially on the *Crista nuchae*; deep to the insertion of *M. omocephalicus Pars superficialis*. The thin *M. rhomboideus cervicis* originates from the *Processus spinosi* of C3 to C7 and from the contralateral muscle in the dorsal midline. *M. rhomboideus thoracis* is continuous with *M. rhomboideus cervicis* and originates from the *Procc. spinosi* of T1 to T7 as well as from the contralateral muscle. The fibres of *M. rhomboideus capitis* run caudo‐ventrally, and the muscle inserts on the *Angulus cranialis*, the medial aspect of the most cranial part of *Margo dorsalis* of the *Scapula* (Figure 2b,c) and on the cranial aspect of the insertion of *M. rhomboideus cervicis*. *M. rhomboideus cervicis* inserts on the medial aspect of the *Angulus cranialis*, *Margo dorsalis* and the *Cartilago scapulae* (Figure 2b,c). *M. rhomboideus thoracis* continues caudally from the cervical rhomboid muscle and inserts on the medial aspect of *Margo dorsalis*, the scapular cartilage and the lateral aspect of *Angulus caudalis* (Figure 2a,c). The muscle fibres of *M. rhomboideus cervicis* and *M. rhomboideus thoracis* run transversely. *M. rhomboideus capitis* and *M. rhomboideus cervicis* will protract the limb by elevating *Angulus cranialis*, whereas *M. rhomboideus thoracis* will retract the limb by elevating the caudal angle. *M. rhomboideus*, acting as a whole, will elevate the *Scapula*.

#### 3.1.5 *M. serratus ventralis cervicis* and *M. serratus ventralis thoracis* (Figure 2b,c) (Tables 1 and S1)

*M. serratus ventralis cervicis* (Alternative names: *M. levator anguli scapulae*/*M. levator scapulae*/*M. serratus ventralis colli*) and *M. serratus ventralis thoracis* (Alternative names: *M. serratus magnus*/*M. serratus anterior*) are fused and form a large, fan‐shaped muscle medial to the *Scapula*. *M. serratus ventralis cervicis* originates on *Procc. transversi* of C3 to C7 and inserts on the cranial half of the *Facies serrata*, ventral to the insertions of *M. rhomboideus capitis* and *M. rhomboideus cervicis* (Figure 2b,c). The thicker *M. serratus ventralis thoracis* originates dorsally to *M. scalenus dorsalis*, on the mid‐lateral surfaces of ribs 1–7 and on the costo‐chondral junctions of ribs 4–7, where the muscle interdigitates with *M. obliquus externus abdominis*. Insertion is on the caudal half of the *Facies serrata*, ventral to the insertion of *M. rhomboideus thoracis* (Figure 2c). Contraction of *M. serratus ventralis cervicis* will protract the limb, while contraction of *M. serratus ventralis thoracis* will retract the limb. *M. serratus ventralis thoracis* may also act as a muscle of inspiration by pulling the ribs cranially.

#### 3.1.6 *M. brachiocephalicus* (Figures 1a, 3a–c, and 4a,b) (Tables 1 and S1) (alternative name: *M. cephalohumeralis*)

**FIGURE 3:**
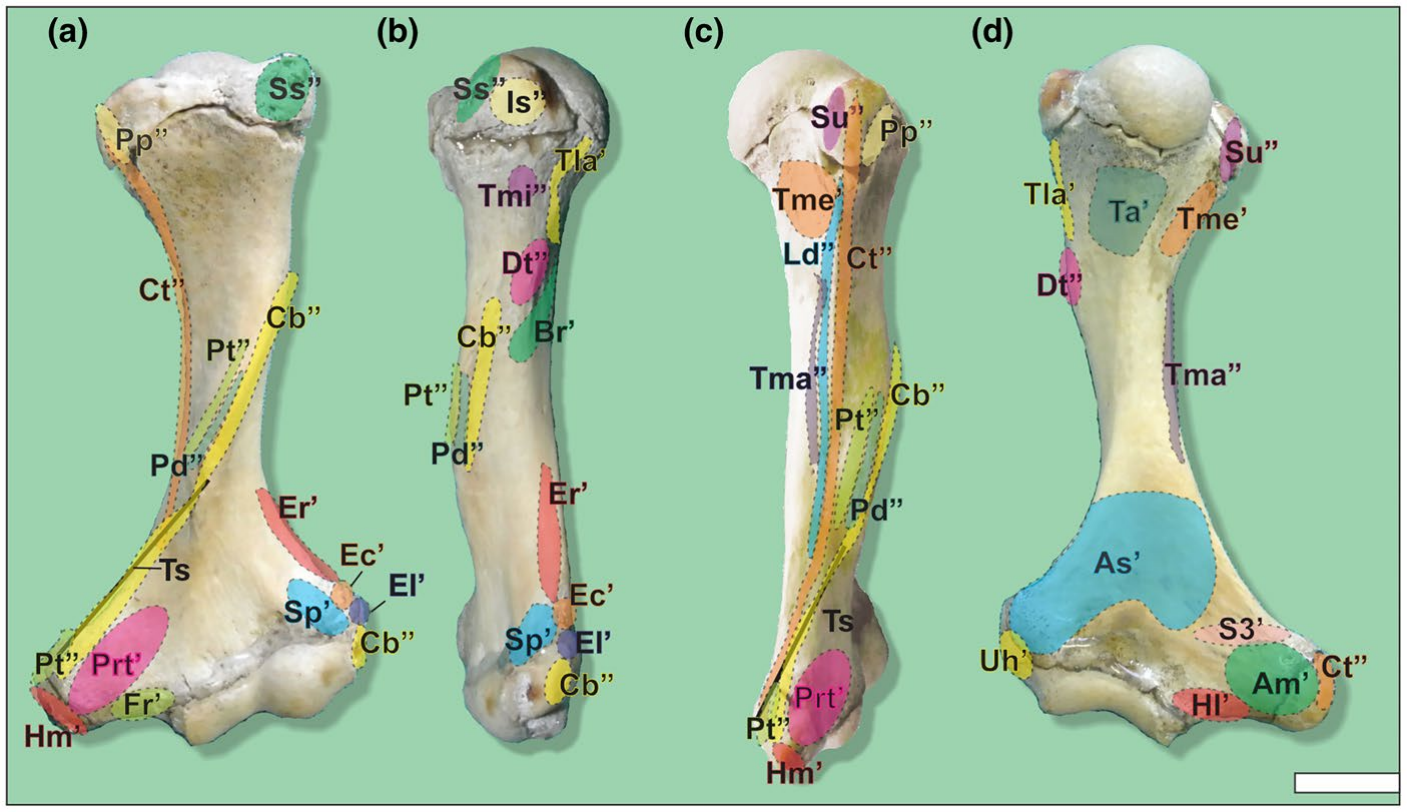
Sites of muscle origin (′) and insertion (″) on the left *Humerus* of a 7.3 kg Temminck's pangolin; (a) Cranial view; (b) Lateral view; (c) Medial view; (d) Caudal view. (a) *M. cleidobrachialis* (Cb), *M. cutaneus trunci* (Ct), *M. extensor carpi radialis* (Er), *M. extensor digitorum communis* (Ec), *M. extensor digitorum lateralis* (El), *M. flexor carpi radialis* (Fr), *M. flexor digitorum profundus Caput humerale Venter medialis* (Hm), *M. pectoralis descendens* (Pd), *M. pectoralis profundus* (Pp), *M. pectoralis transversus* (Pt), *M. pronator teres* (Prt), *M. supinator* (Sp) and *M. supraspinatus* (Ss). The solid black line (Ts) indicates the tendinous structure spanning the distal *Crista humeri* and *Epicondylus medialis*. (b) *M. brachialis* (Br), *M. deltoideus* (Dt), *M. infraspinatus* (Is), *M. teres minor* (Tmi) and *M. triceps brachii Caput lateralis* (Tla). Remaining annotations are as follows: (a). (c) *M. latissimus dorsi* (Ld), *M. subscapularis* (Su), *M. teres major* (Tma) and *M. triceps brachii Caput medialis* (Tme). Remaining annotations as for (a and b). (d) *M. anconeus* (As), *M. anconeus medialis* (Am), *M. flexor digitorum profundus Caput humerale Venter lateralis* (Hl), *M. flexor digitorum superficialis Venter digiti III* (S3), *M. extensor carpi ulnaris Caput humerale* (Uh) and *M. triceps brachii Caput accessorium* (Ta). Remaining annotations as for (a–c). Bar = 1 cm.

**FIGURE 4:**
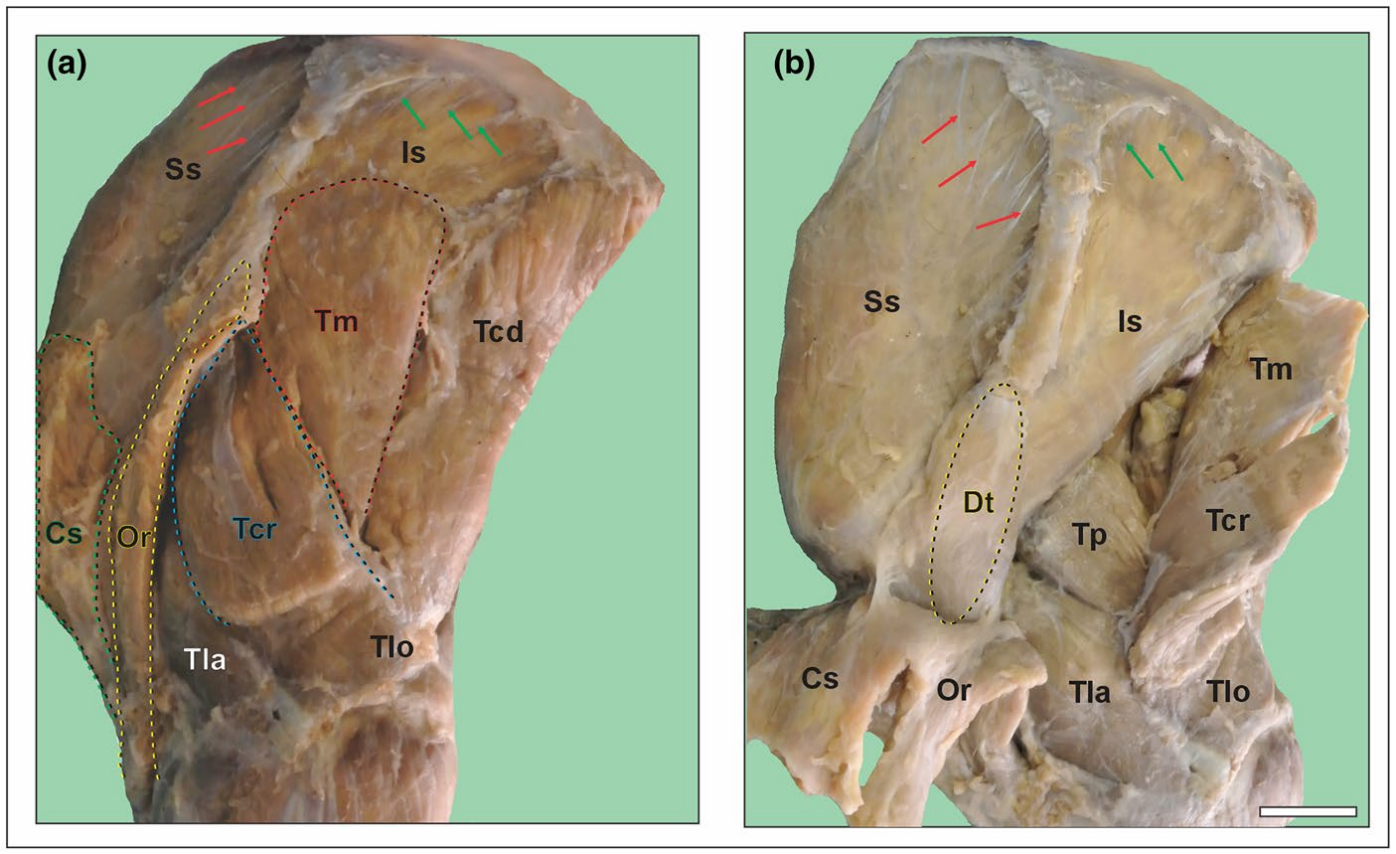
Lateral view of the left upper thoracic limb of a 7.0 kg Temminck's pangolin: (a) Superficial view (the aponeurosis covering the *M. infraspinatus* has been removed); (b) Deep view. (a) *M. cleidocephalicus* (Cs), *M. infraspinatus* (Is) displaying numerous internal tendons (green arrows), *M. omoradialis* (Or), *M. supraspinatus* (Ss) displaying numerous internal tendons (red arrows), *M. triceps brachii Caput lateralis* (Tla), *M. triceps brachii Caput longum* (Tlo) with *Venter caudalis* (Tcd) and *Venter medius* (Tm). (b) *M. deltoideus* (Dt), *M. triceps brachii Caput longum Venter cranialis* (Tcr) and *Venter profundus* (Tp). Remaining annotations as for (a). Bar = 1 cm.

*M. brachiocephalicus* is a broad, strap‐like muscle that lies superficially in the lateral neck and shoulder region and in the cranio‐lateral brachium (Figure 1a). The muscle is composed of *M. cleidocephalicus* (Figure 4a,b) (which only consists of a *Pars cervicalis*—see Discussion) and *M. cleidobrachialis*. Both muscles originate on the *Intersectio clavicularis* (*Clavicula* that has been reduced to a thin band of fibrous tissue palpable in some specimens) situated cranial to the shoulder joint. The muscle fibres of *M. cleidocephalicus* run cranio‐dorsally. The caudal margin is fused to the cranial aspect of *M. trapezius Pars cervicalis*. *M. cleidocephalicus* inserts on the full extent of the *Crista nuchae* of the skull, superficial to the insertion of *M. omocephalicus* and *M. omomastoideus*, as well as on the thin median raphe of the neck at the level of vertebrae C1 and C2. No *Pars mastoidea* was observed in the specimens studied. The muscle fibres of *M. cleidobrachialis* run caudo‐ventrally and fuse with *M. omoradialis* over the cranio‐lateral brachium (see Section 3.2.3) as well as to the *Tuberculum majus* of the *Humerus* via fascia. *M. cleidobrachialis* inserts along the length of the *Crista humeri*, onto the tendinous structure that runs between the distal edge of the *Crista humeri* and the *Epicondylus medialis*, onto the cranio‐medial part of the *Epicondylus medialis*, and, via a strong fibrous tendon that crosses *M. omoradialis*, onto the distal *Epicondylus lateralis* (Figure 3a–c). Both muscles effectively form a muscle chain that functions as a unit to protract the limb by drawing the distal part of the *Humerus* cranially.

#### 3.1.7 *M. cutaneus trunci* (Figures 1a, 3a,c,d, and 5a,b) (Tables 1 and S1) (alternative names: *M. panniculus carnosus*/*M. brachiolateralis*/*M. abdominohumeralis*)

**FIGURE 5:**
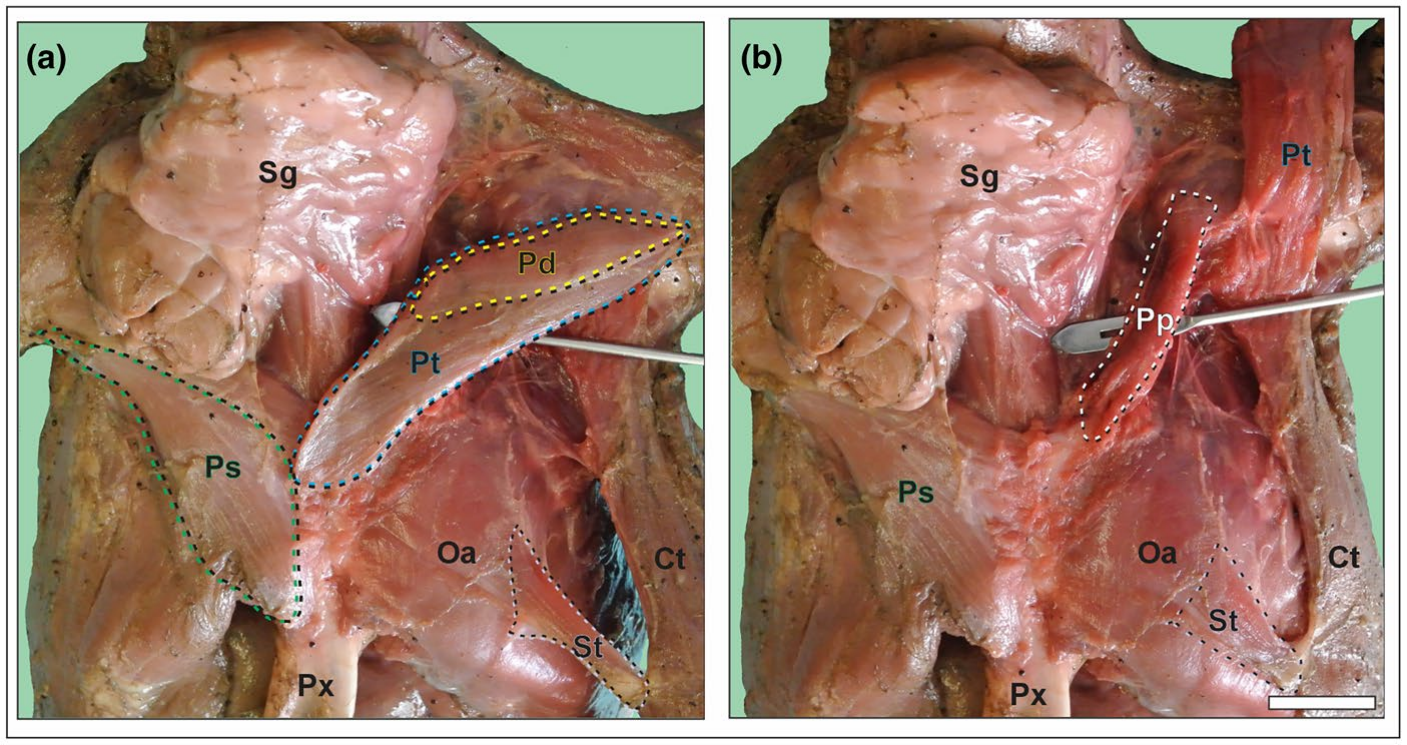
Ventral view of the thorax of a 3.0 kg Temminck's pangolin: (a) Superficial view; (b) Deep view. (a) Left *M. cutaneus trunci* (Ct), left *M. obliquus externus abdominus* (Oa), left *M. pectoralis descendens* (Pd, undermined), right *Mm. pectorales superficiales* (Ps, the components have not been exposed), left *M. pectoralis transversus* (Pt, undermined), left *M. rectus sternalis* (St), *Processus xiphoideus* (Px) and left salivary gland (Sg) reflected cranio‐medially. (b) The left *M. pectoralis transversus* (Pt) has been reflected cranially to reveal the *M. pectoralis profundus* (Pp). Remaining annotations as for (a). Bar = 2 cm.

*M. cutaneus trunci* is an extensive, thick muscle that covers the lateral aspect of the thorax and abdomen and is the most superficial muscle on the trunk (Figures 1a and 5a,b). The dorsal extent of this muscle is the lateral edge of the row of scales in the dorsal midline. The ventral extent of the muscle is the most ventral longitudinal row of scales. The muscle originates from the dermis with multiple thin tendinous structures that are attached to the base of the scales over the lateral aspect of the trunk. The muscle runs horizontally with some muscle fibres attaching to the lateral surfaces of ribs 3–6, just ventral to the origin of *M. serratus ventralis thoracis*. *M. cutaneus trunci* inserts via a short, broad tendon on the *Tuberculum minus*, the *Crista tuberculi minoris* and, medial to *M. latissimus dorsi* (see Section 3.1.8) and *M. teres major* (see Section 3.2.5), on the *Tuberositas teres major* (Figure 3a,c). Some distal muscle fibres are fused to fibres of *M. pectoralis transversus* (see Section 3.1.9). Additionally, a long, thin tendon inserts onto the tendinous structure that runs between the *Crista humeri* and the *Epicondylus medialis* (Figure 3a,c) as well as on the caudal part of the *Epicondylus medialis* (Figure 3d). A thin muscle fascicle develops from the cranio‐lateral aspect of *M. cutaneus trunci*, crosses the superficial surface of *M. triceps brachii Caput longum* (see Section 3.3.1) and inserts on the caudal margin of *M. omoradialis* over the lateral brachium (see Section 3.2.3). The muscle pulls the base of the scales down tightly towards the skin surface and assists in adopting a defensive posture through ventral flexion of the body, due to its broad origin. It will also retract the limb by drawing the *Humerus* caudally.

#### 3.1.8 *M. latissimus dorsi* (Figures 1a,b and 3c) (Table S1)

*M. latissimus dorsi* is a flat, broad, roughly triangular muscle that covers two‐thirds of the lateral surface of the thorax up to the level of the costo‐chondral junctions. It lies deep to *M. cutaneus trunci* (Figure 1a,b). This muscle originates via four muscle bundles from the lateral surfaces of ribs 7–11 (attributing a serrated appearance), and a thin aponeurosis over the dorsal thoracic area. As the muscle fibres run cranio‐ventrally, the muscle increases in thickness and divides to form two sheets. The superficial muscle sheet is fused to the distal part of *M. teres major* (see Section 3.2.5) and inserts via a long, broad tendon on the *Tuberositas teres major* (distal to the insertion of *M. teres major*) (Figure 3c), while the deep muscle sheet inserts along the length of the *Crista tuberculi minoris* and on the *Tuberositas teres major* (Figure 3c). *M. latissimus dorsi* will retract the limb by drawing the *Humerus* caudally.

#### 3.1.9 *Mm. pectorales superficiales* (Figures 3a–c and 5a,b) (Tables 1 and S1) (alternative name: *M. pectoralis major*)

*Mm. pectorales superficiales* are well‐developed muscles that form the ventral connection between the trunk and the thoracic limbs and consist of *M. pectoralis descendens* and the much larger *M. pectoralis transversus* (Figure 5a,b). These two muscles display varying degrees of fusion. *M. pectoralis descendens* originates on the cranial half of the *Corpus sterni* and the contralateral muscle in the ventral midline, whereas the deeper *M. pectoralis transversus* originates from the entire *Corpus sterni*, from the base of the elongated *Processus xiphoideus* and the contralateral muscle. The muscle fibres run laterally, and both muscles decrease in width towards their insertions. *M. pectoralis descendens* inserts on the ventral part of the *Crista humeri* (Figure 3a–c) and *M. pectoralis transversus* inserts on the ventral part of the *Crista humeri* and the proximo‐medial part of the *Epicondylus medialis* (Figure 3a–c). Both muscles will adduct the limb, and *M. pectoralis transversus* will either protract the limb if the limb is retracted, or it will retract the limb if the limb is protracted, as a result of its very broad origin.

#### 3.1.10 *M. pectoralis profundus* (Figures 3a,c and 5b) (Tables 1 and S1) (alternative name: *M. Pectoralis minor*)

*M. pectoralis profundus* is much smaller than *Mm. pectorales superficiales* (Figure 5b). It also contributes to the ventral connection between the trunk and the thoracic limbs. The muscle originates on the *Manubrium sterni*, just lateral to the ventral midline. Its fibres run laterally, and the muscle inserts with a long tendon onto the cranial surface of the *Tuberculum minor* of the *Humerus* (Figure 3a,c). The muscle will adduct the limb.

#### 3.1.11 M. rectus sternalis* (Figure 5a,b) (Tables 1 and S1)

In one specimen, *M. rectus sternalis* was present. This thin, insignificant muscle originates from the ventral margin of *M. cutaneus trunci*. The muscle fibres run cranio‐ventrally, and the insertion is on the ventral fascia and epimysium of *M. obliquus externus abdominis*.

### 3.2 Muscles of the shoulder joint

This group of muscles is arranged around the shoulder joint (*Articulatio humeri*) and originates from the lateral, caudal and medial surfaces of the *Scapula* and inserts on the proximal *Humerus*; exceptions include *M. biceps brachii*, *M. omoradialis* and *M. triceps brachii*. The muscles function as either extensors or flexors of the shoulder joint; some will also tense the shoulder joint capsule. The sequence of the muscles described is based on their origin, position around the *Scapula* and function.

#### 3.2.1 *M. supraspinatus* (Figures 2a, 3a,b, and 4a,b) (Table S2)

*M. supraspinatus* is a stout, oblong muscle that projects beyond the distal part of the cranial surface of the *Scapula* and is visible from the *Facies costalis*. It originates on the *Fossa supraspinatus* (Figure 2a), and there are numerous internal tendons interspersed in the proximal part of the muscle (Figure 4a,b). Muscle fibres attach to the lateral aspect of the shoulder joint capsule. Insertion is on the *Humerus* via a broad tendon on the cranio‐lateral surface of the *Tuberculum majus* (Figure 3a,b). The muscle extends the shoulder joint if the *Humerus* is retracted, tenses the shoulder joint capsule, and the broad tendon of insertion stabilises the joint laterally by acting as a lateral collateral ligament.

#### 3.2.2 *M. infraspinatus* (Figures 1a,b, 2a, 3b, and 4a,b) (Table S2)

*M. infraspinatus* is a triangular‐shaped muscle that is largely covered by an aponeurosis, *M. omoradialis* (see Section 3.2.3) and *M. triceps brachii Caput longum* (see Section 3.3.1) (Figure 1a,b). The muscle originates from the *Fossa infraspinatus* (Figure 2a) and numerous internal tendons are visible in the proximal half of the muscle (Figure 4). Insertion is on the *Facies m. infraspinati* of the *Humerus* (Figure 3b) via a long, well‐developed tendon. The muscle flexes the shoulder joint when the *Humerus* is protracted, and the tendon of insertion acts as a lateral collateral ligament to stabilise the joint laterally.

#### 3.2.3 M. omoradialis* (Figures 1a, 2a, 4a,b, 6a, and 8a,b) (Tables 1 and S2) (alternative name: *M. supinator longus*)

**FIGURE 6:**
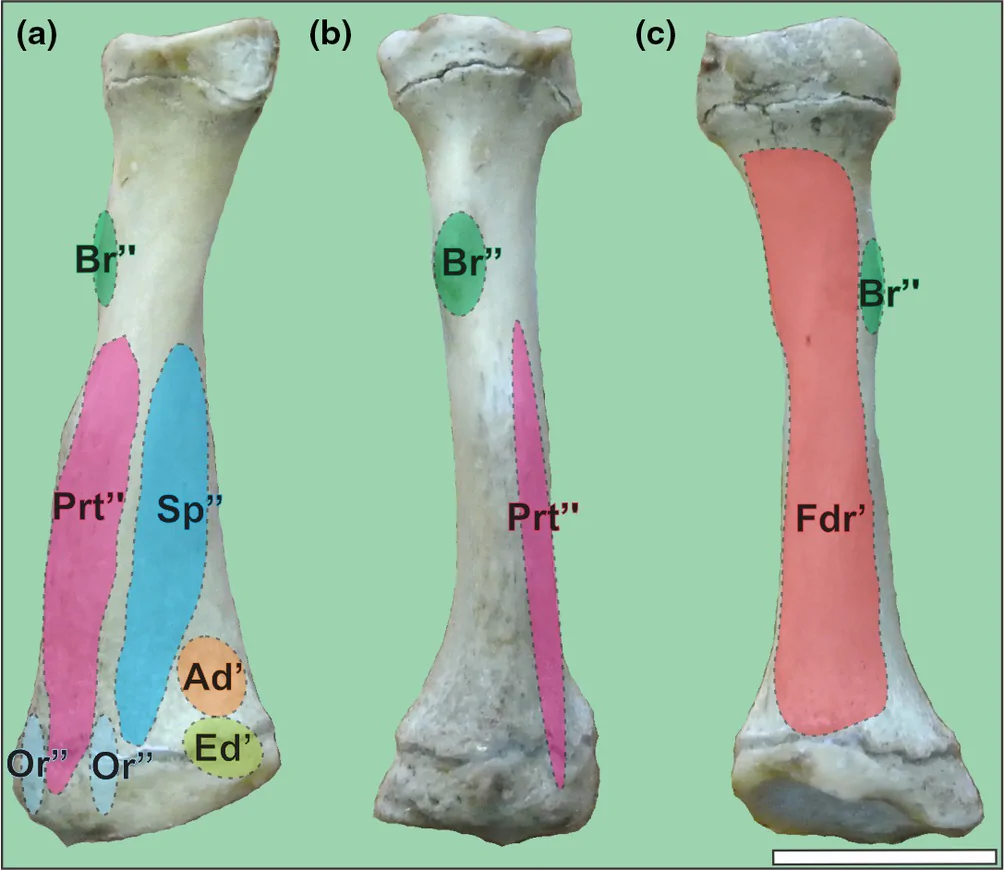
Sites of muscle origin (′) and insertion (″) on the left *Radius* of an 8.3 kg Temminck's pangolin; (a) Cranial view; (b) Medial view; (c) Caudo‐lateral view. (a, b) *M. abductor digiti I longus* (Ad), *M. brachialis* (Br), *M. extensor digiti I et II* (Ed), *M. omoradialis* (Or), *M. pronator teres* (Prt) and *M. supinator* (Sp). (c) *M. brachialis* (Br) and *M. flexor digitorum profundus Caput radiale* (Fdr). Bar = 1 cm.

This muscle (named here *M. omoradialis*, see Discussion and Table 1) is a long, thin muscle that occurs superficially on the lateral to cranio‐lateral aspect of the thoracic limb, where it spans both the shoulder and elbow joints (Figures 1a, 4a,b and 8a,b). The proximal part is more fibrous, and the distal part is fleshier. The muscle originates caudo‐distally on the *Spina scapulae* and *Acromion* (Figure 2a) as well as from the aponeurosis covering *M. infraspinatus*. As the muscle fibres cross the lateral aspect of the shoulder joint, the cranio‐medial margin of the proximal *M. omoradialis* is attached to the caudo‐lateral margin of *M. deltoideus* (see Section 3.2.4) by strong, thick fascia. *M. omoradialis* and surrounding muscles are contained through thick fascia over the cranial surface of the brachium. The muscle crosses the flexor surface of the elbow, superficial to *M. extensor carpi radialis* (see Section 3.5.1), passes over the oblique tendon of *M. abductor digiti I longus* (see Section 3.5.8), and is attached to the fascia of the antebrachium (Figure 8a,b). A long, relatively broad tendon, which is fused to the *Fascia dorsalis manis*, inserts on the most distal part of the cranio‐medial *Radius*, on either side of the groove on the *Trochlea radii* (Figure 6a). The muscle will simultaneously flex the shoulder and elbow joints.

#### 3.2.4 *M. deltoideus* (Figures 2a,b, 3b,d, and 4b) (Table S2)

*M. deltoideus* is a small, spindle‐shaped muscle on the lateral aspect of the shoulder joint that lies deep to *M. omoradialis* (Figure 4b). It originates cranio‐ventrally on the *Acromion* (Figure 2a,b). The muscle crosses the distal part of *M. infraspinatus* and the caudolateral aspect of the shoulder joint. Insertion is via a short tendon on the *Tuberositas deltoideus* of the *Humerus* (Figure 3b,d). The muscle flexes the shoulder joint by retracting the *Humerus*.

#### 3.2.5 *M. teres major* (Figures 2c and 3c,d) (Table S2)

*M. teres major* is a fleshy muscle situated caudal to the *Scapula* and is fused to *M. triceps brachii Caput longum* (see Section 3.3.1). The muscle originates on the medial aspect of the *Margo caudalis*, from the *Angulus caudalis* to the *Tuberculum infraglenoidale*, medial to the origin of *M. triceps brachii Caput longum* (Figure 2c). As the muscle fibres travel ventrally, some attach to the distal part of *M. latissimus dorsi* and some to the caudal surface of the shoulder joint capsule. The muscle inserts with a short, broad tendon on the ventral part of the *Crista tuberculi minoris* as well as on the dorsal part of the *Tuberositas teres major* of the *Humerus* (Figure 3c,d). The tendons of insertion of *M. latissimus dorsi* and *M. teres major* are fused to some degree. *M. teres major* flexes the shoulder joint and tenses the joint capsule.

#### 3.2.6 *M. teres minor* (Figures 2a and 3b) (Table S2)

*M. teres minor* is a small muscle that lies caudal to the distal part of the *Scapula* and is partly covered by *M. infraspinatus*. The muscle originates on the *Tuberculum infraglenoidale* (Figure 2a), lateral to the origin of *M. triceps brachii Caput longum Venter profundus* (see Section 3.3.1). The muscle fibres run ventrally and cross the caudo‐lateral surface of the shoulder joint, where they attach to the joint capsule. Insertion is on the *Tuberositas teres minor* on the proximal *Humerus* (Figure 3b) via a relatively long tendon. The muscle will flex the shoulder joint and tense the joint capsule.

#### 3.2.7 *M. subscapularis* (Figures 2b,c and 3c,d) (Table S2)

*M. subscapularis* is a flat, triangular muscle with a thick and shiny epimysium on the medial aspect of the *Scapula*. The muscle originates on the *Fossa subscapularis* (Figure 2b,c). The fibres run ventrally, are interspersed with numerous internal tendons, and attach to the medial surface of the shoulder joint capsule. *M. subscapularis* inserts with a broad tendon caudo‐medially on the *Tuberculum minus* of the *Humerus* (Figure 3c,d). The tendon acts as a medial collateral ligament to stabilise the shoulder joint. The muscle will play a minor role in tensing the shoulder joint capsule and extending, flexing or adducting the shoulder joint if the *Humerus* is retracted, protracted or abducted, respectively.

### 3.3 Muscles of the elbow joint

Some of the muscles acting on the elbow joint (*Articulatio cubiti*), such as *M. triceps brachii Caput longum* and *M. biceps brachii*, originate on the *scapula* and thus also act on the shoulder joint. However, most of the muscles originate from the *humerus* and function solely on the elbow joint. The muscles insert on the proximal *radius* and *ulna*. The main function will be extension or flexion of the elbow joint; some will also tense the elbow joint capsule. The sequence of the muscles described is according to their function.

#### 3.3.1 *M. triceps brachii* (Figures 1a,b, 2a,c, 3b–d, 4a,b, and 7b–d) (Table S3)

**FIGURE 7:**
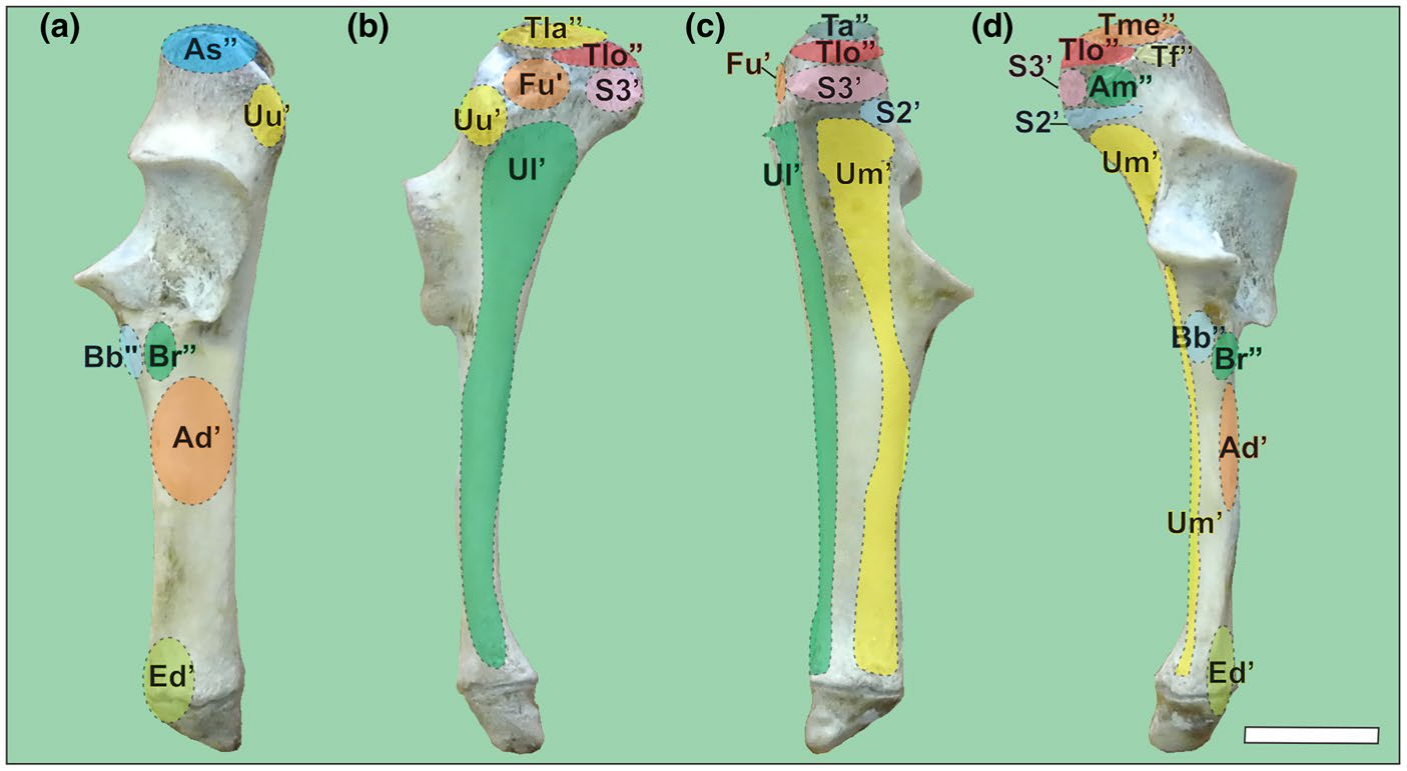
Sites of muscle origin (′) and insertion (″) on the left *Ulna* of an 8.3 kg Temminck's pangolin; (a) Cranial view; (b) Lateral view; (c) Caudal view; (d) Medial view. (a) *M. abductor digiti I longus* (Ad), *M. anconeus* (As), *M. biceps brachii* (Bb), *M. brachialis* (Br), *M. extensor carpi ulnaris Caput ulnare* (Uu) and *M. extensor digiti I et II* (Ed). (b) *M. flexor carpi ulnaris* (Fu), *M. flexor digitorum profundus Caput ulnare Venter lateralis* (Ul), *M. flexor digitorum superficialis Venter digiti III* (S3), *M. triceps brachii Caput lateralis* (Tla) and *M. triceps brachii Caput longum* (Tlo). Remaining annotations as for (a). (c) *M. flexor digitorum profundus Caput ulnare Venter medialis* (Um), *M. flexor digitorum superficialis Venter digiti II* (S2) and *M. triceps brachii Caput accessorium* (Ta). Remaining annotations as for (a) and (b). (d) 4″—*M. anconeus medialis* (Am), *M. tensor fasciae antebrachia* (Tf) and *M. triceps brachii Caput medialis* (Tme). Remaining annotations as for (a–c). Bar = 1 cm.

*M. triceps brachii* is the largest muscle of the thoracic limb and lies in the triangular space formed between the *Scapula*, *Humerus* and *Olecranon* (Figures 1a,b and 4a,b). The muscle is composed of four heads (*Caput longum*/*lateralis/medialis/accessorium*), which display varying degrees of fusion. The *Caput longum* is, in turn, composed of four bellies, named here according to the position of their origins on the *Scapula* relative to each other. The four bellies additionally display varying degrees of fusion. The *Venter cranialis* originates caudo‐distally from the *Spina scapulae* and caudally on the *Acromion* (Figure 2a), deep to the origin of *M. omoradialis*. The *Venter medius* displays an aponeurotic origin overlying *M. infraspinatus* (Figure 4). The *Venter caudalis* originates laterally on the *Margo caudalis*, spanning the *Angulus caudalis* to the *Tuberculum infraglenoidale* (Figure 2a), and is positioned lateral to the origin of *M. teres major*. Lastly, the *Venter profundus* originates on the *Tuberculum infraglenoidale* (Figure 2c), medial to the origin of *M. teres minor*. The remaining three heads originate from the *Humerus*. The *Caput lateralis* originates from the *Linea m. tricipitis* (Figure 3b,d) and its muscle fibres are fused to the fibres of the deeper *M. anconeus* (see Section 3.3.3). The *Caput medialis* originates from the caudo‐medial humeral neck, the medial aspect of the proximal *Humerus* and from the *Crista tuberculi minoris* (Figure 3c,d). The *Caput accessorium* originates from the caudal aspect of the humeral neck (Figure 3d) as well as from the caudal aspect of the shoulder joint capsule. *M. triceps brachii* displays an extremely broad tendon of insertion on the *Tuber olecrani*; the *Caput longum* occupies two‐thirds of the width of the caudal *Tuber olecrani* (Figure 7b–d); the *Caput lateralis/medialis/accessorium* insert proximo‐laterally (Figure 7b), proximo‐medially (Figure 7d) and mid‐proximally (Figure 7c), respectively, deep to the insertion of the *Caput longum*. *M. triceps brachii* is a powerful extensor of the elbow joint. Additionally, the *Caput longum* will flex the shoulder joint and tense the shoulder joint capsule.

#### 3.3.2 *M. tensor fasciae antebrachii* (Figure 7d) (Tables 1 and S3) (alternative names: *M. dorso‐epitrochlien* */* *M. latissimo‐olecranalis*)

*M. tensor fasciae antebrachii* is a small, flat muscle that originates from the epimysium on the ventral surface of the superficial muscle sheet of *M. latissimus dorsi*. The muscle inserts proximo‐medially on the *Tuber olecrani* (Figure 7d) (medial to the insertion of *M. triceps brachii Caput longum*) as well as on the antebrachial fascia. The muscle tenses the antebrachial fascia and extends the elbow joint.

#### 3.3.3 *M. anconeus* (Figures 3d and 7a) (Tables 1 and S3) (alternative name: *M. anconeus externus*)

*M. anconeus* is a well‐developed muscle that covers the *Fossa olecrani* and lies deep to *M. triceps brachii, Caput lateralis/accessorium*. The origin is broad and includes the *Epicondylus lateralis*, *Crista supracondylaris lateralis*, the dorsal rim of the *Fossa olecrani*, as well as the *Crista supracondylaris medialis* (Figure 3d). As the muscle covers the caudal aspect of the elbow joint capsule, its fibres attach to the capsule. The muscle inserts cranially on the proximal *Olecranon* (Figure 7a). The muscle extends the elbow joint and tenses the joint capsule.

#### 3.3.4 M. anconeus medialis* (Figures 3d, 7d, and 9b) (Tables 1 and S3) (alternative names: *M. epitrochleo‐anconeus*/*M. anconeus internus*/*M. epitrochleo‐olecranalis*/*M. triceps brachii caput mediale accessorium*)

This muscle (named here *M. anconeus medialis*, see Discussion and Table 1) is a short, fleshy muscle on the medial aspect of the elbow joint (Figure 9b). It originates caudally on the *Epicondylus medialis* (Figure 3d) and inserts medially on the *Tuber olecrani*, distal to the insertion of *M. triceps brachii* (Figure 7d). The epimysium of the ventral surface of the muscle partly serves as the origin of *M. flexor digitorum superficialis Venter digiti II* (see Section 3.5.9). *M. anconeus medialis* will extend the elbow joint.

#### 3.3.5 *M. biceps brachii* (Figures 2a–c, 7a,d, and 8c,d) (Table S3)

**FIGURE 8:**
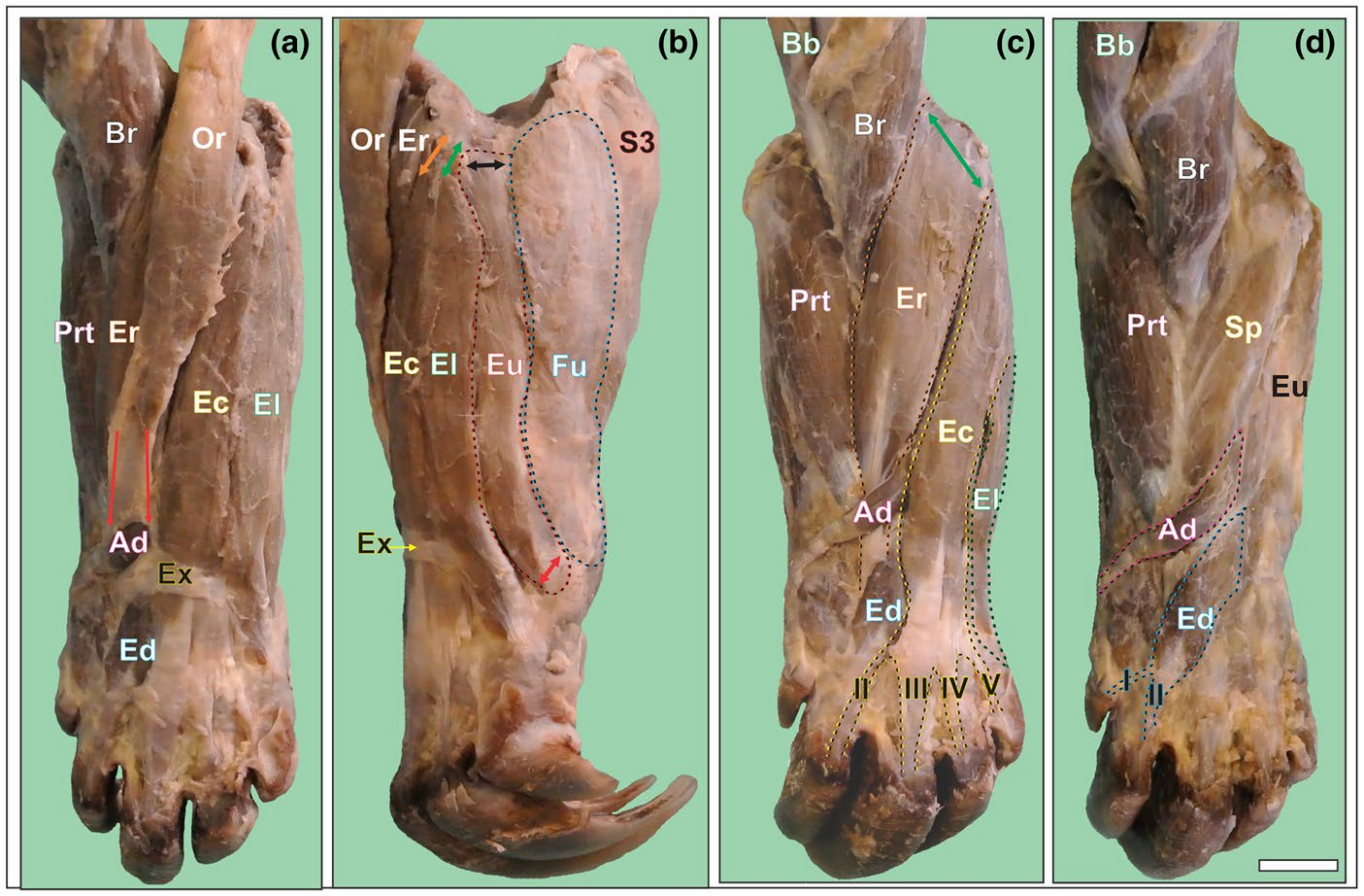
Cranial view of the left *Antebrachium* of a 7.3 kg (a) and a 7.0 kg (b, c) Temminck's pangolin: (a) Superficial view; (b) Lateral view; (c) Superficial view with the *M. omoradialis* removed; (d) Deep view. (a) *M. abductor digiti I longus* (Ad), *M. brachialis* (Br), *M. extensor carpi radialis* (Er), *M. extensor digiti I et II* (Ed), *M. extensor digitorum communis* (Ec), *M. extensor digitorum lateralis* (El), *M. omoradialis* (Or), which inserts with a tendon on either side (red arrows) of the groove of the *Trochlea radii*, *M. pronator teres* (Prt) and *Retinaculum extensorum* (Ex). (b) *M. extensor carpi ulnaris* (Eu), *M. flexor carpi ulnaris* (Fu) and *M. flexor digitorum superficialis Venter digiti III* (S3). Remaining annotations as for (a). (c) *M. biceps brachii* (Bb) and *M. extensor digitorum communis* tendons of insertion to digits II, III, IV and V. The double‐headed green arrow indicates the broad origin of the *M. extensor carpi radialis*. Remaining annotations as for (a) and (b). (d) *M. extensor digit I et II* (Ed) tendons of insertion to digits I and II, and *M. supinator* (Sp). Remaining annotations as for (a–c). Bar = 1 cm.

*M. biceps brachii* is a spindle‐shaped muscle that lies on the cranio‐medial surface of the brachium and spans the shoulder and elbow joints. The muscle originates via a long tendon on the *Tuberculum supraglenoidale* of the *Scapula* (Figure 2a–c), and the tendon of origin is held in the *Sulcus intertubercularis* by the thickened cranial shoulder joint fascia. A synovial bursa, the *Bursa intertubercularis*, surrounds the tendon of origin. This bursa is an extension of the shoulder joint capsule. As the muscle travels distally, it passes deep to the tendinous structure running between the *Crista humeri* and the *Epicondylus medialis* (graphically indicated on Figure 3a,c). The muscle inserts with a single tendon (Figure 8c,d) on the medial surface of the proximal *Ulna* (Figure 7a,d), medial to the insertion of *M. brachialis* (see Section 3.3.6). The muscle flexes the elbow joint and extends the shoulder joint.

#### 3.3.6 *M. brachialis* (Figures 3b, 6a–c, 7a,d, and 8a,c,d) (Tables 1 and S3) (alternative name: *M. Brachialis anticus*)

*M. brachialis* is a fleshy muscle that runs in the *Sulcus m. brachialis* (brachial groove) of the *Humerus*. It originates with a broad tendon proximo‐laterally on the *Humerus* distal to the *Linea m. tricipitis* (proximal part of the brachial groove, also known as the radial groove) (Figure 3b). As it travels distally, it is attached to the lateral surface of the *Crista humeri*. The muscle inserts with a broad tendon on the *Tuberositas radii* (Figure 6a–c) as well as on the cranio‐medial surface of the proximal *Ulna* (Figure 7a,d), just lateral to the insertion of *M. biceps brachii*. *M. brachialis* is a strong flexor of the elbow joint.

### 3.4 Muscles of the radio‐ulnar joints

Rotational movement between the *Radius* and *Ulna* is possible in *S*. *temminckii*. The muscles that perform these actions originate on the distal *Humerus* and insert on the *Radius* only. The function of these muscles would be either pronation or supination, as well as flexion of the elbow joint.

#### 3.4.1 *M. pronator teres* (Figures 3a,c, 6a,b, and 8a,c,d) (Table S4)

*M. pronator teres* is an extremely well‐developed muscle that lies on the cranio‐medial aspect of the antebrachium (Figure 8a,c,d), cranial to *M. flexor carpi radialis* (see Section 3.5.2), where the two apposing surfaces are fused. It originates from the entire cranial surface of the *Epicondylus medialis* of the *Humerus*, lateral to the insertions of *M. pectoralis transversus* and the distal part of *M. cleidobrachialis* (Figure 3a,c). As the muscle fibres run cranio‐laterally, the deep surface of the muscle is fused to *M. flexor digitorum profundus Caput radialis* (see Section 3.5.10). The extensive insertion is along the entire length of the crest on the *Facies cranialis* of the *Radius* (Figure 6a,b), immediately medial to the insertion of *M. supinator* (see Section 3.4.2). The muscle will predominantly pronate both the proximal and distal radio‐ulnar joints and secondarily flex the elbow joint.

#### 3.4.2 *M. supinator* (Figures 3a,b, 6a, and 8d) (Tables 1 and S4) (alternative name: *M. Supinator brevis*)

*M. supinator* is a large, triangular muscle that lies deep to *M. extensor carpi radialis* (see Section 3.5.1) and the proximal part of *M. extensor digitorum communis* (see Section 3.5.5) on the cranio‐lateral aspect of the antebrachium (Figure 8d). The muscle originates from the cranio‐lateral surface of the distal *Epicondylus lateralis* (Figure 3a,b), from the lateral collateral ligament of the elbow joint as well as from the distal cranio‐lateral surface of the elbow joint capsule. There is a small sesamoid bone embedded in the tendon of origin. The muscle fibres run cranio‐medially. The insertion is wide, fleshy and along the length of the crest on the *Facies cranialis* of the *Radius*, up to the distal physeal line (Figure 6a). The muscle will predominantly supinate the proximal and distal radio‐ulnar joints as well as flex the elbow joint and tense the elbow joint capsule.

### 3.5 Muscles of the carpal and digital joints

The long muscles that activate the carpal (*Articulatio carpal*) and digital joints (*Articulationes interphalangeae*) are arranged around the *Radius* and *Ulna* and function mainly as either extensors or flexors. Most of the extensor muscles of the carpus and digits originate from the *Epicondylus lateralis* of the *Humerus*, while the flexor muscles originate from the *Epicondylus medialis* of the *Humerus*. This group of muscles that act on both the carpal and digital joints all have fleshy origins, with the exception of *M. flexor carpi radialis*, *M. flexor carpi ulnaris* and *M. extensor digitorum communis* and display noticeable tendons of insertion. The sequence of muscles described below follows the main joints which they affect.

#### 3.5.1 *M. extensor carpi radialis* (Figures 3a,b, 8a–c, and 10a) (Table S5)

*M. extensor carpi radialis* is a fleshy muscle that lies on the cranio‐lateral surface of the antebrachium, deep to *M. omoradialis* (Figure 8a–c). The muscle displays a wide, fleshy origin on the *Crista supracondylaris lateralis* of the *Humerus* (Figure 3a,b). As the muscle travels distally, it passes deep to *M. abductor digit I longus* (see Section 3.5.8) and *M. extensor digiti I et II* (see Section 3.5.7) in the distal antebrachium. The tendon of insertion crosses the dorsal aspect of the *Carpus*, where it is surrounded by a synovial sheath, and is secured over the *Carpus* by the *Retinaculum extensorum* (Figure 8a,b). Insertion is on the *Tuberositas ossis metacarpalis III* (Figure 10a). The insertion is cranial to the carpal joint's axis of rotation, and the muscle will extend the carpal joint. As a result of its relatively proximal origin on the *Humerus*, the muscle may also flex the elbow joint.

#### 3.5.2 *M. flexor carpi radialis* (Figures 3a and 10a,b) (Table S5)

*M. flexor carpi radialis* is a thin muscle on the medial antebrachium. The muscle lies between *M. pronator teres* and the medial belly of both the radial head and humeral head of *M. flexor digitorum profundus* (see Section 3.5.10) and is fused to all of them. It originates distally from the cranio‐lateral surface of the humeral *Epicondylus medialis* (Figure 3a). The muscle splits into two branches just proximal to the *Articulatio antebrachiocarpea*; the lateral branch inserts onto the medio‐palmar aspect of the palmar carpal cartilage covering the palmar carpal joint capsule, while the medial branch passes over the medio‐palmar aspect of the *Carpus*, palmar to the *Canalis carpi* and inserts proximally on the medial projection of *Os carpale I* (Figure 10a,b). The insertion is caudal to the carpal joint's axis of rotation, and the muscle flexes the carpal joint. The muscle also tenses the carpal joint capsule.

#### 3.5.3 *M. extensor carpi ulnaris* (Figures 3d, 7a,b, 8b,d, and 10b) (Table S5)

*M. extensor carpi ulnaris* is a fleshy muscle on the lateral antebrachium, positioned between *M. extensor digitorum lateralis* (see Section 3.5.6) and *M. flexor carpi ulnaris* (see Section 3.5.4) (Figure 8b,d). The muscle displays a broad, fleshy origin on the caudo‐lateral part of the distal *Epicondylus lateralis* (Figure 3d) as well as on the cranio‐lateral *Olecranon* (Figure 7a,b). The two heads, *Caput humerale* and *Caput ulnare*, are mainly fused, and the *Caput ulnare* lies deep to *M. flexor carpi ulnaris*. As the muscle fibres run distally, they are attached to the lateral surface of *M. extensor digitorum lateralis* and the disto‐lateral part of the *Corpus ulnae*. The muscle crosses the lateral aspect of the carpus and inserts via a short, broad tendon palmaro‐laterally on the *Basis* of *Os metacarpale V* (Figure 10b) and occasionally also with a thin fascial band onto the lateral side of the *Os carpi accessorium*. The tendon of insertion does not possess a synovial sheath. The insertion is caudal to the carpal joint's axis of rotation, and the muscle will flex the carpal joint.

#### 3.5.4 *M. flexor carpi ulnaris* (Figures 7b,c, 8b, 9a, and 10b) (Table S5)

**FIGURE 9:**
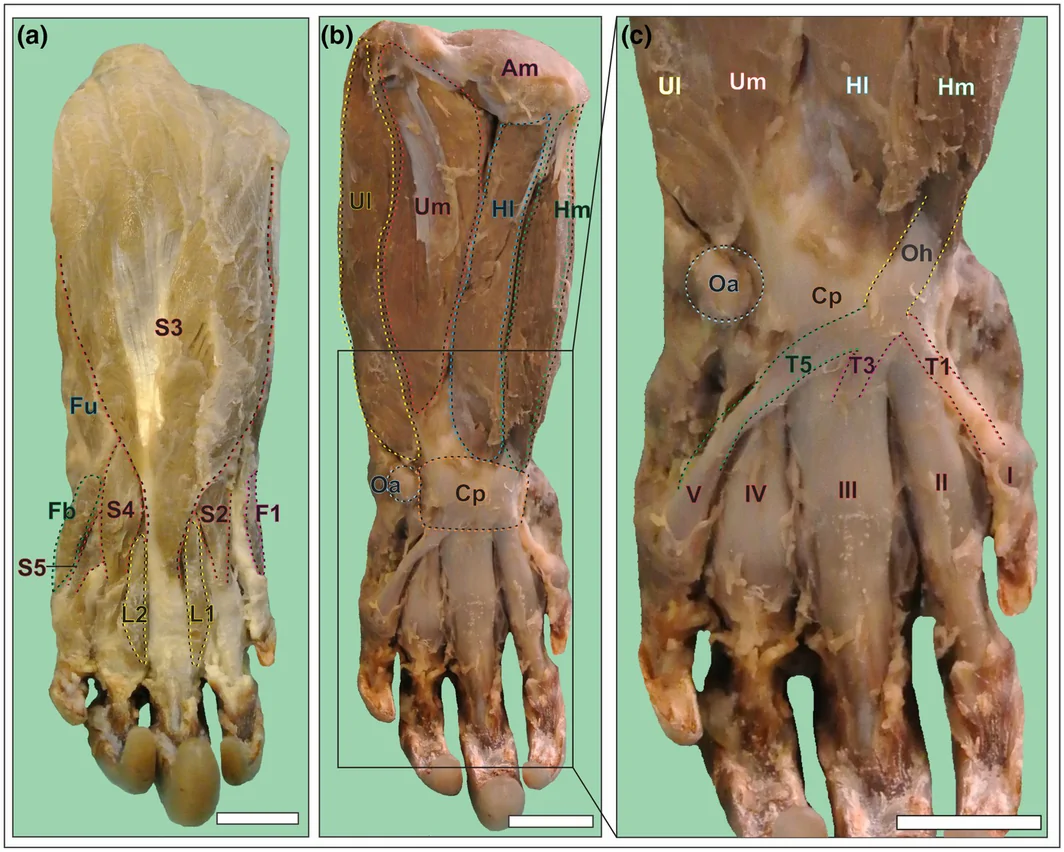
Palmar view of the left distal limb of a 7.0 (a) and 7.3 kg (b, c) Temminck's pangolin: (a) Superficial view; (b) The *M. flexor digitorum superficialis* is removed; (c) Carpal region enlarged. (a) *M. flexor digitorum brevis* (Fb), *M. flexor carpi ulnaris* (Fu), *M. flexor digiti I brevis* (F1), medial *M. lumbricalis* (L1) and middle *M. lumbricalis* (L2), *M. flexor digitorum superficialis Venter digiti II* (S2), *Venter digiti III* (S3), *Venter digiti IV* (S4) and *Venter digiti V* (S5). (b) *M. anconeus medialis* (Am), Carpal plate (Cp) of the *M. flexor digitorum profundus* tendon of insertion, *M. flexor digitorum profundus Caput humerale Venter lateralis* (Hl), *Caput humerale Venter medialis* (Hm), *Caput ulnare Venter lateralis* (Ul), *Caput ulnare Venter medialis* (Um) and *Os carpi accessorium* (Oa, outlined for clarity). (c) Oblique tendon (Oh) of the *Caput humerale Venter medialis* (Hm), branches to digits 1 (T1), 3 (T3) and 5 (T5) and the *M. flexor digitorum profundus* tendons of insertion to digits I–V. Remaining annotations as for (b). Bar = 1 cm.

**FIGURE 10:**
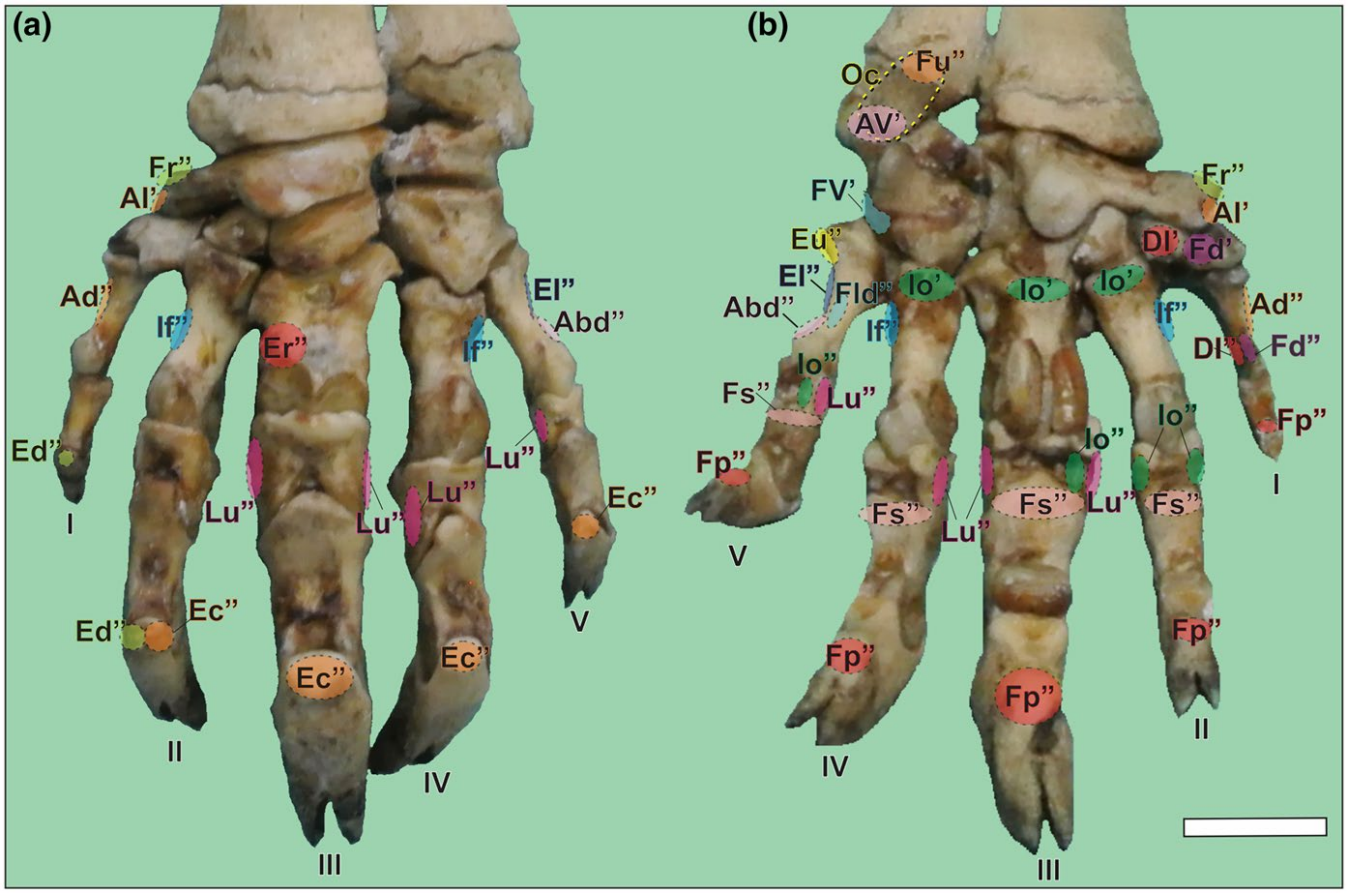
Sites of muscle origin (′) and insertion (″) on an articulated left *Manus* of an 8.3 kg Temminck's pangolin; (a) Dorsal view; (b) Palmar view. (a) *M. abductor digiti V* (Abd), *M. abductor digiti I longus* (Ad), *M. abductor digiti I brevis* (AI), *M. extensor digitorum communis* (Ec), *M. extensor digiti I et II* (Ed), *M. extensor digitorum lateralis* (El), *M. extensor carpi radialis* (Er), *M. flexor carpi radialis* (Fr), *Mm. interflexorii* (If) and *Mm. lumbricales* (Lu). (b) *M. adductor digiti I* (DI), *M. extensor carpi ulnaris* (Eu), *M. flexor digiti I brevis* (Fd), *M. flexor digiti V* (Fld), *M. flexor digitorum profundus* (Fp), *M. flexor digitorum superficialis* (Fs), *M. flexor carpi ulnaris* (Fu) and *Mm. interossei* (Io). The *Os carpi accessorium* (Oc) is outlined for clarity. Remaining annotations as for (a). Bar = 1 cm.

*M. flexor carpi ulnaris* is a thin, flat, semi‐fibrous muscle situated on the caudo‐lateral antebrachium, between *M. extensor carpi ulnaris*, where it partially covers the muscle and is fused to it, and *M. flexor digitorum superficialis Venter digiti III* (see Section 3.5.9) (Figures 8b and 9a). The muscle originates laterally on the proximal *Olecranon* (Figure 7b,c) and the muscle fibres are fused to the underlying *M. flexor digitorum profundus Caput ulnare* (see Section 3.5.10). Insertion is proximal on the *Os carpi accessorium* (Figure 10b) via a short tendon which does not possess a synovial sheath. The muscle inserts caudal to the carpal joint's axis of rotation and will flex the carpal joint.

#### 3.5.5 *M. extensor digitorum communis* (Figures 3a,b, 8a–c, and 10a) (Table S5)

*M. extensor digitorum communis* is a fleshy muscle on the cranio‐lateral aspect of the antebrachium, between *M. extensor carpi radialis* and *M. extensor digitorum lateralis* (Figure 8a–c). The muscle originates cranio‐laterally on the *Epicondylus lateralis* (Figure 3a,b) via a short, thin tendon. The proximal muscle fibres are fused to *M. extensor digitorum lateralis* and *M. supinator*. The well‐developed *Retinaculum extensorum* secures the broad tendon of insertion over the distal antebrachium and dorsal carpus. The tendon is surrounded by a synovial sheath over the *Trochlea radii* and carpal region. At the distal end of the carpus, the tendon splits into two branches, which in turn divide to insert on digits II–V (Figure 8c). The larger medial branch inserts predominantly on the *Processus extensorius* of the *Phalanx distale* of digit III, and sends a thin tendon to the *Proc. extensorius* of digit II (Figures 8c and 10a). The smaller lateral branch inserts mainly on the *Proc. extensorius* of digit IV, and sends a small tendon to the *Proc. extensorius* of digit V (Figures 8c and 10a). The muscle will extend the carpal joint as well as the metacarpophalangeal, proximal interphalangeal and distal interphalangeal joints of digits II–V.

#### 3.5.6 *M. extensor digitorum lateralis* (Figures 3a,b, 8a–c, and 10a,b) (Tables 1 and S5) (alternative names: *M. abductor digiti V longus*/*M. extensor annularis*/*M. extensor digiti minimi)*

*M. extensor digitorum lateralis* lies cranio‐laterally on the antebrachium, between *M. extensor digitorum communis* and *M. extensor carpi ulnaris* (Figure 8a–c). The muscle displays a short, thin origin from the most cranio‐lateral aspect of the *Epicondylus lateralis* (Figure 3a,b), disto‐lateral to the origin of *M. extensor digitorum communis*. The origin and proximal muscle fibres are fused to *M. extensor digitorum communis* and *M. extensor carpi ulnaris*. As the muscle fibres run distally, they attach along the latero‐distal part of the *Ulna*. The tendon of insertion does not possess a synovial sheath and crosses the cranio‐lateral aspect of the *Carpus*. Insertion is proximally on the *Margo lateralis* of *Os metacarpale V* (Figure 10a,b). A small tendon branches off and joins the deep surface of the *M. extensor digitorum communis* tendon to digit IV. The muscle will extend the carpometacarpal joint of digit V and, via the *M. extensor digitorum communis* tendon, the metacarpophalangeal, proximal interphalangeal and distal interphalangeal joints of digit IV. The muscle may also abduct digit V.

#### 3.5.7 *M. extensor digiti I et II* (Figures 6a, 7a,d, 8a,c,d, and 10a) (Tables 1 and S5) (alternative name: *M. extensor indicis*)

*M. extensor digiti I et II* is a small muscle that lies obliquely over the most cranio‐distal part of the antebrachium and cranial surface of the carpal joint (Figure 8a,c,d). The proximal muscle fibres are partly covered by, and fused to, *M. extensor digitorum communis* and *M. extensor digitorum lateralis*. The broad, fleshy origin lies on the cranio‐lateral *Trochlea radii* (Figure 6a) and the cranio‐medial *Caput ulnae* (Figure 7a,d). As the muscle travels disto‐medially, it lies superficial to the tendon of insertion of *M. extensor carpi radialis*. The tendon of insertion is covered by a synovial sheath over the *Carpus* and is attached to the dorso‐medial surface of the carpal joint capsule. The tendon divides into two branches over the dorsal aspect of the proximal phalanges (Figure 8d) and inserts on the *Processus extensorius* of digit I and medial to the insertion of *M. extensor digitorum communis*, on digit II (Figure 10a). The muscle extends the carpal, metacarpophalangeal and distal interphalangeal joints of digit I as well as the carpal, metacarpophalangeal, proximal interphalangeal and distal interphalangeal joints of digit II.

#### 3.5.8 *M. abductor digiti I longus* (Figures 6a, 7a,d, 8a,c,d, and 10a,b) (Tables 1 and S5) (alternative names: *M. extensor ossis metacarpi pollicis*, *M. extensor pollicis primus*)

*M. abductor digiti I longus* lies obliquely over the cranial antebrachium, between *M. supinator* and *M. extensor digiti I et II* (Figure 8a,c,d). The muscle is partially covered by *M. omoradialis*, *M. extensor digitorum communis* and *M. extensor digitorum lateralis*. The broad, fleshy origin is on the cranial surface of the proximal half of the *Corpus ulnae* (Figure 7a,d) as well as distally on the cranio‐lateral *Corpus radii* (Figure 6a), distal to the insertion of *M. supinator*. As the muscle tapers disto‐medially, it passes over the *M. extensor carpi radialis* tendon in the *Trochlea radii*. A synovial sheath surrounds the tendon of insertion as it passes over the *M. extensor carpi radialis* and carpal joint area. The tendon inserts proximally on the *Margo medialis* of *Os metacarpale I* (Figure 10a,b) and in some specimens on *Os metacarpale II*. The muscle abducts the digit/s as the insertion is on the abaxial surface of metacarpus I (and II). The muscle will also extend the carpal joint.

#### 3.5.9 *M. flexor digitorum superficialis* (Figures 3d, 7b–d, 9a, and 10b) (Tables 1 and S5) (alternative names: *M. flexor digitorum sublimis*)

*M. flexor digitorum superficialis* is the most superficial muscle on the caudal aspect of the antebrachium (Figure 9a) and lies directly beneath the skin, caudo‐medial to *M. flexor carpi ulnaris*. The muscle consists of four bellies that each supply a single tendon to either digit II, III, IV or V. There is no tendon of insertion that supplies digit I. The muscle bellies are fused to varying degrees as well as to the underlying *M. flexor digitorum profundus*, and there is variation in the origins and positions of these bellies. The muscle belly to digit III (*Venter digiti III*) is the most superficial and originates proximal to the origin of *M. anconeus medialis* on the caudal aspect of the *Epicondylus medialis* (Figure 3d) as well as distal to the insertion of *M. triceps brachii* on the caudal aspect of the *Olecranon* (Figure 7b–d). The broad, flat *Venter digiti III* continues distally to insert on digit III. The muscle belly to digit IV (*Venter digiti IV*) originates from the deep surface of the digit III muscle belly in the mid‐antebrachium. The thin *Venter digiti IV* travels distally, deep to the *Venter digiti III* and inserts on digit IV. The small muscle belly that supplies digit V (*Venter digiti V*) originates distally from the deep muscle fibres of the digit IV belly in the metacarpus. The thin tendon travels disto‐laterally to insert on digit V. It may also originate from the palmar aspect of the tendon of the belly of digit III in the proximal metacarpus and passes disto‐laterally, over the tendon of digit IV, to insert on digit V. The muscle belly to digit II (*Venter digiti II*) originates from the ventral epimysium of *M. anconeus medialis* as well as the caudo‐medial aspect of the *Olecranon* (Figure 7c,d). This belly lies deep to the belly of digit IV and is fused to the underlying *M. flexor digitorum profundus*. The *Venter digiti II* continues distally and inserts on digit II. *M. flexor digitorum superficialis* lies palmar to the *Canalis carpi*, and the tendons of insertion form over the distal carpal region. The tendons each share a synovial sheath with the corresponding tendon of *M. flexor digitorum profundus* from the mid metacarpus distally. Each tendon of insertion is covered by the *Ligamentum anulare palmare* over the metacarpophalangeal joint. Distal to the anular palmar ligament, each tendon forms a sleeve, the *Manicum flexorum*, to allow *M. flexor digitorum profundus* tendons to pass distally. The tendons of insertion are each covered by the *Ligamentum anulare digiti proximale* of the corresponding digit. The individual tendons insert on either side of the *M. flexor digitorum profundus* tendons on the *Tuberositas flexoria* of the middle phalanges of digits II‐V (Figure 10b). Flexion of the carpal joint as well as the metacarpophalangeal and proximal interphalangeal joints of digits II‐V occurs.

#### 3.5.10 *M. flexor digitorum profundus* (Figures 3a,c,d, 6c, 7b–d, 9b,c, and 10b) (Table S5)

*M. flexor digitorum profundus* is an extremely well‐developed muscle that lies deep to *M. flexor digitorum superficialis* on the caudal aspect of the antebrachium (Figure 9b,c). The origin of the three heads, the *Caput humerale*, *Caput ulnare* and *Caput radiale*, which are fused to varying degrees, is broad. The *Caput humerale* and *Caput ulnare* are composed of both medial and lateral bellies (Figure 9b,c). The *Venter medialis* of the *Caput humerale* (Alternative name: *M. flexor digitorum profundus humerale mediale*) originates distally on the cranio‐medial *Epicondylus medialis* (Figure 3a,c) and from the caudal aspect of the elbow joint capsule. The *Venter lateralis* (Alternative name: *M. flexor digitorum profundus humerale laterale*) originates distally on the caudo‐lateral *Epicondylus medialis* (Figure 3d) and from the elbow joint capsule. The *Venter medialis* of the *Caput ulnare* originates from the caudo‐medial and medial aspect of the *Olecranon*, the caudal aspect of the elbow joint capsule and from the entire caudo‐medial *Corpus ulnae* (Figure 7c,d). The *Venter lateralis* originates from the caudo‐lateral and lateral aspect of the *Olecranon* and from the entire lateral and caudo‐lateral *Corpus ulnae* (Figure 7b,c). The *Caput radiale* originates deep to the *Caput humerale* from the entire caudal and caudo‐lateral *Collum radii* and *Corpus radii* (Figure 6c) and from the elbow joint capsule. As the muscle fibres of all the heads travel distally, they fuse into a broad tendon plate over the palmar carpal area (Figure 9b,c) (referred to here as the carpal plate), deep to the *Retinaculum flexorum*, from where the tendons of insertion branch to the individual digits. The medial belly of the *Caput humerale* contributes to the disto‐medial carpal plate and forms a disto‐lateral oblique tendon on the palmar aspect of the carpal plate (Figure 9c). The oblique tendon from the medial belly of the *Caput humerale* divides into three and contributes to digits I, III and V (Figure 9c). The lateral belly of the *Caput humerale* contributes to the medial side of the palmar aspect of the carpal plate and its main contributions are to digits II and III (Figure 9c). The *Caput ulnare* attaches to the lateral and dorso‐lateral aspect of the carpal plate and contributes mainly to digit IV and a small part to digit III (Figure 9c). The *Caput radiale* contributes to the deep aspect of the dorsal part of the carpal plate. A synovial sheath surrounds the carpal plate over the carpus from the level of the *Os carpi accessorium* to the proximal metacarpus. Each tendon of insertion travels distally, deep to the corresponding tendon of *M. flexor digitorum superficialis*. *M. flexor digitorum profundus* tendon to digit I is not covered by *M. flexor digitorum superficialis*. The tendons are held in position by the corresponding *Ligamentum anulare digiti distale* distal to the insertion of *M. flexor digitorum superficialis*. Insertion is on the *Tuberculum flexorium* of the distal phalanges of all five digits (Figure 10b). The muscle is a strong flexor of the carpal, metacarpophalangeal and distal interphalangeal joints of digit I as well as the carpal, metacarpophalangeal, proximal interphalangeal and distal interphalangeal joints of digits II‐V. The muscle will also tense the elbow joint capsule.

### 3.6 Muscles of the forepaw and individual digits

The short muscles of the metacarpophalangeal joints are arranged either between the digital flexor tendons or lie on the palmar aspect of the metacarpal and digital bones. They function as flexors of the metacarpophalangeal and digital joints. The individual muscles of digits I and V will either flex, abduct or adduct the individual digits.

#### 3.6.1 *Mm. interflexorii* (Figure 10a,b) (Table S6)

*Mm. interflexorii* are two small muscles of the manus that lie over the palmar metacarpal area. The medial and lateral muscles originate superficially on the distal aspect of *M. flexor digitorum superficialis* muscle bellies to digits II and IV, respectively, over the carpo‐metacarpal region (not shown graphically). *Mm. interflexorii* are short and insert on the proximal *Margo medialis* of *Os metacarpale II* and the proximal *Margo lateralis* of *Os metacarpale IV*, respectively (Figure 10a,b). The muscles flex the metacarpophalangeal joints of digits II and IV.

#### 3.6.2 *M. flexor digitorum brevis* (Figure 9a) (Table S6)

*M. flexor digitorum brevis* is a small muscle of the manus that lies over the palmar metacarpus (Figure 9a), superficial to *M. abductor digiti V* (see Section 3.6.8). The muscle originates from the disto‐lateral aspect of the *M. flexor digitorum superficialis* muscle belly to digit V in the distal carpus, as well as from the fascia of the *Os carpi accessorium*. Insertion is latero‐palmarly on the metacarpophalangeal joint capsule of digit V. This muscle will flex the metacarpophalangeal joint of digit V.

#### 3.6.3 *Mm. lumbricales* (Figures 9a and 10a,b) (Table S6)

*Mm. lumbricales* are the deepest group of small muscles on the palmar manus (Figure 9a). The three muscles originate on the palmar surface of the tendons of insertion of *M. flexor digitorum profundus* in the distal carpus and decrease in size from medial to lateral. The most medial muscle inserts medially on the body of the proximal phalanx of digit III (Figure 10a,b). The middle muscle divides, and the smaller belly inserts laterally on the body of the proximal phalanx of digit III while the larger belly inserts axially on the body of the proximal phalanx of digit IV (Figure 10a,b). The lateral muscle inserts axially on the body of digit V's proximal phalanx (Figure 10a,b). The action will be to flex the metacarpophalangeal joints of digits III–V.

#### 3.6.4 *Mm. interossei* (Figure 10b) (Table S6)

*Mm. interossei* are relatively well‐developed muscles of the manus that lie on the palmar aspect of the metacarpal bones. The four muscles originate palmarly from the bases of *Ossa metacarpalia II‐IV* (Figure 10b) as well as from the disto‐palmar aspect of the thickened carpal joint capsule. The muscles run obliquely and display numerous internal tendons. Two muscles from MC II insert palmarly on the axial and abaxial surfaces of the proximal phalangeal body of digit II, one muscle from MC III inserts medio‐palmarly on the proximal phalangeal body of digit III, and one muscle from MC IV inserts on the axial surface of the proximal phalangeal body of digit V (Figure 10b). There is no insertion on digit IV. The *Mm*. *interossei* will flex the metacarpophalangeal joints II, III and V as well as tense the carpal joint capsule. The insertion of the muscles may suggest a degree of abduction and adduction.

#### 3.6.5 *M. abductor digiti I brevis* (Figure 10a,b) (Table S6)

*M. abductor digiti I brevis* is a small, thin muscle that originates distally on the medial projection of *Os carpale I* (Figure 10a,b) and inserts on the medial aspect of the metacarpophalangeal joint capsule of digit I. This muscle abducts digit I.

#### 3.6.6 *M. flexor digiti I brevis* (Figures 9a and 10b) (Table S6)

*M. flexor digiti I brevis* is a relatively well‐developed muscle (Figure 9a) that originates palmarly on *Os carpale I* and inserts disto‐palmarly on the body of *Os metacarpale I* (Figure 10b). The muscle flexes digit I.

#### 3.6.7 *M. adductor digiti I* (Figure 10b) (Table S6)

*M. adductor digiti I* is a small muscle that originates latero‐palmarly on *Os carpale I* (Figure 10b). Insertion is disto‐laterally on the body of *Os metacarpale I* (Figure 10b). The muscle adducts the first digit.

#### 3.6.8 *M. abductor digiti V* (Figure 10a,b) (Tables 1 and S6) (alternative: *M. abductor minimi digiti*)

*M. abductor digiti V* is a well‐developed muscle that originates palmarly on the disto‐lateral surface of the *Os carpi accessorium* (Figure 10b). The muscle inserts distally on the *Margo lateralis* of *Os metacarpale V* (Figure 10a,b) and abducts the digit.

#### 3.6.9 *M. flexor digiti V* (Figure 10b) (Tables 1 and S6) (alternative: *M. flexor brevis minimi digiti*)

*M. flexor digiti V* is a small muscle that originates palmaro‐lateral on the disto‐palmar aspect of *Os carpale IV* (Figure 10b). Insertion is distal on the *Facies palmaris* of *Os metacarpale V* (Figure 10b). The muscle will flex digit V.

## 4 DISCUSSION

### 4.1 General features and nomenclature

The muscle maps generated in this study, based on the osteology of *S*. *temminckii* (Steyn et al., 2018), provided a deeper understanding of the various muscle functions. Similar to other morphological studies that incorporate muscle maps (Dunn et al., 2022; Ercoli et al., 2015; Marshall et al., 2021), this work also constitutes a valuable resource for understanding and reconstructing the frontlimb musculature of several extinct pangolin genera, such as *Necromanis*, *Cryptomanis*, *Patriomanis* and *Eomanis* (Gaudin et al., 2009). Similarly, muscle maps generated for the *P*. *tigris* frontlimb are equally important in plotting muscles in related fossil taxa (Dunn et al., 2022).

The thoracic limb myology of *S*. *temminckii* was generally similar (for differences see below) to that reported in other pangolin species, including *P. tricuspis*, *P. tetradactyla*, *M. pentadactyla* and *M. javanica* (Humphry, 1869; Kawashima et al., 2015; Windle & Parsons, 1899). The above‐mentioned species also shared many similarities with other myrmecophagous and fossorial mammals, such as *M*. *tridactyla*, *T*. *tetradactyla* (Humphry, 1869; Windle & Parsons, 1899), and *O. afer* (Endo et al., 2002; Galton, 1869; Thewissen & Badoux, 1986). However, the evaluation and comparison of literature on thoracic limb myology of fossorial and myrmecophagous mammals is challenging as several of the studies, which span 146 years (Dellon, 1986; Galton, 1869, 1874; Humphry, 1869; Kawashima et al., 2015; O'Driscoll et al., 1991; Peterka, 1936; Windle & Parsons, 1899), implemented a variety of anatomical terms (which were either antiquated or based on human terminology) other than the official terminology according to *Nomina Anatomica Veterinaria* (N. A. V.). Additionally, some muscles identified in *S*. *temminckii* have not been named in N. A. V. Clarification of the terminology used in the above studies compared with the present investigation is presented in Table 1.

In mammals, *M. brachiocephalicus* consists of *M. cleidobrachialis* and *M. cleidocephalicus*, with the latter being subdivided into *Pars cervicalis, Pars mastoidea* and *Pars occipitalis* (ICVGAN, 2017). *M. deltoideus Pars clavicularis* in humans is the analogue of *M. cleidobrachialis* in mammals where the clavicle has been reduced (ICVGAN, 2017). A variable combination of these muscles has been observed in different species. *M. cleidobrachialis*, as well as the *Pars cervicalis* (sometimes simply named *M. cleidocephalicus*) and *Pars mastoidea* (sometimes named *M. cleidomastoideus*), are observed in a variety of carnivores such as *C*. *familiaris* (Evans & de Lahunta, 2013), *G*. *cuja* (Ercoli et al., 2015), *L*. *pardalis* (Julik et al., 2012), *L*. *gymnocercus* (de Souza Junior et al., 2018) and *P*. *tigris* (Dunn et al., 2022). A separate *Pars occipitalis* is not described in these species. In Xenarthrans, such as armadillos and sloths, only the *Pars mastoidea* of *M. cleidocephalicus* is observed, and the *Pars cervicalis* and *Pars occipitalis* are consistently absent (Marshall et al., 2021; Tucker et al., 2026). While *M. cleidobrachialis* is present in sloth species (Tucker et al., 2026), this muscle is not observed in Cingulata (Marshall et al., 2021). In *S*. *temminckii*, *M. cleidocephalicus* consists only of a *Pars cervicalis* (as defined in carnivore literature (Ercoli et al., 2015; Evans & de Lahunta, 2013; Julik et al., 2012)), as well as *M. cleidobrachialis*. A *Pars mastoidea* is not observed.

In myrmecophagous mammals lacking a *Clavicula*, *M. deltoideus Pars clavicularis* was described as being continuous with *M. trapezius* to form *M. cephalohumeralis* (Windle & Parsons, 1899). *M. brachiocephalicus* was not reported in *M*. *pentadactyla*, and *M. trapezius* was described as a large muscle that took origin from both the *Crista nuchae* and the median raphe of the neck, dorsal to the spinous processes of the cervical vertebrae (Humphry, 1869; Kawashima et al., 2015). The muscle inserted partially on the *Spina scapulae*, but most of the muscle mass continued distally and inserted on the *Crista humeri* and *Epicondylus medialis* of the *Humerus*, as well as on a tendinous band that runs between the *Crista humeri* and the *Epicondylus medialis* (Humphry, 1869; Kawashima et al., 2015). Based on the above description and the observation in the present study that the caudal margin of *M. brachiocephalicus* and the cranial margin of *M. trapezius Pars cervicalis* were fused in *S*. *temminckii*, it would appear that *M. trapezius* described by Humphry (1869) and Kawashima et al. (2015), and *M. cephalohumeralis* described by Windle and Parsons (1899), represents the fused *M. brachiocephalicus* and *M. trapezius Pars cervicalis* in *S*. *temminckii*. Thus, contrary to earlier reports, *M*. *pentadactyla* most likely possesses an *M. brachiocephalicus*.

An *M. occipitoscapularis* and *M. mastoscapularis* have been described in *M*. *pentadactyla* (Humphry, 1869; Kawashima et al., 2015), and both muscles were classified as either segments of *M. trapezius* (Humphry, 1869) or as accessory scapular levator muscles (Kawashima et al., 2015). The latter usually attach to the *Margo cranialis* of the *Scapula*, but as a result of adaptations to a fossorial lifestyle, the attachment of the accessory scapular levator muscles in *M*. *pentadactyla* is modified to attach to the *Spina scapulae* and therefore provide stabilisation of the *Scapula* (Kawashima et al., 2015). According to the practice of naming a muscle from its origin towards its insertion (Gadow, 1882), *M. occipitoscapularis* would originate on the *Os occipitale* and insert on the *Scapula* (Humphry, 1869; Kawashima et al., 2015). Following convention, the site of origin of any muscle is firstly defined as either being on the less mobile part or, secondly, on the more proximal part of the body, as compared to the site of insertion, which is either on the more mobile part or the more distal part (Gadow, 1882). The naming of *M. occipitoscapularis* in *M*. *pentadactyla* (Kawashima et al., 2015) would better reflect naming convention if it were termed *M*. omocephalicus, indicating an origin from the *Scapula* which is attached to the less mobile trunk, and insertion on the head. The term *M. omocephalicus* was thus used for the similar muscle in *S*. *temminckii* (see Table 1). Similarly, *M. mastoscapularis* (Humphry, 1869; Kawashima et al., 2015) might be better termed *M. omomastoideus*, which indicates an origin on the *Scapula* and an insertion on the *Processus mastoideus* of the *Os temporale*. The term *M. omomastoideus* was therefore used in *S*. *temminckii* (see Table 1). While *M. omotransversarius* originates on the acromion of the *Scapula*, the insertion differs in species. In carnivores, the muscle consistently inserts on the second cervical vertebra (de Souza Junior et al., 2018; Ercoli et al., 2015; Evans & de Lahunta, 2013; Julik et al., 2012). In sloth species, the muscle may be absent or, if present, it may insert either on the first cervical vertebra or the mastoid process (Tucker et al., 2026). In Cingulata, *M. omotransversarius* attaches to the base of the cranium or mastoid process (Marshall et al., 2021). Although *M. omotransversarius* is not described in Pholidota (Humphry, 1869; Kawashima et al., 2015, present study), the combined *M. omocephalicus* and *M. omomastoideus* in the present study similar to *M. occipitoscapularis* and *M. mastoscapularis* in *M*. *pentadactyla* (Humphry, 1869; Kawashima et al., 2015), may perform a similar role to the typical *M. omotransversarius*. The extended insertion of *M. omocephalicus* and *M. omomastoideus* may allow for more vigorous ventral flexion of the head during the defensive posture of the pangolin.

*M. supinator longus*, more commonly known as *M. brachioradialis* (ICVGAN, 2017; Hermanson, 2013), has been described in *M*. *tridactyla*, *T*. *tetradactyla* (Windle & Parsons, 1899), and *O*. *afer* (Galton, 1869; Humphry, 1869). This muscle has also been described in *P*. *tricuspis* (Windle & Parsons, 1899), *M*. *pentadactyla* (Humphry, 1869; Kawashima et al., 2015; Windle & Parsons, 1899) and *M*. *javanica* (Windle & Parsons, 1899). In the above‐mentioned pangolin species, however, it was noted that *M. supinator longus* originated on the *Spina scapulae* and *Acromion* (Humphry, 1869; Kawashima et al., 2015; Windle & Parsons, 1899), while it originated on the distal *Humerus* of Vermilingua and Tubulidentata (Galton, 1869; Windle & Parsons, 1899). As *M. brachioradialis* is named according to its origin and insertion (Gadow, 1882), this muscle (termed *M. supinator longus*), as previously described in Pholidota species (Humphry, 1869; Kawashima et al., 2015; Windle & Parsons, 1899), is not comparable to *M. brachioradialis* (also termed *M. supinator longus*) in Vermilingua and Tubulidentata (Galton, 1869; Windle & Parsons, 1899). Based on the origin of this muscle, the more appropriate term *M. omoradialis* was used in *S*. *temminckii* to avoid confusion. It is suggested that this term may also be more appropriate for describing this specific muscle in all Pholidota species.

A variety of names have been ascribed to *M. anconeus medialis* (see Supplementary Table 7). This muscle was described as characteristic of the myrmecophagous mammal group previously known as the Edentata (Galton, 1874; Windle & Parsons, 1899). The muscle has been reported in *P*. *tetradactyla* (Windle & Parsons, 1899), *P*. *tricuspis* (Windle & Parsons, 1899), *M*. *pentadactyla* (Humphry, 1869; Windle & Parsons, 1899) and *M*. *javanica* (Windle & Parsons, 1899). Additionally, it was also present in *O*. *afer* (Galton, 1869, 1874), *B*. *tridactylus* (Galton, 1874; Humphry, 1869), *Bradypus variegatus* (brown‐throated three‐toed sloth) (Olson et al., 2018), *M*. *tridactyla*, *T*. *tetradactyla* (Galton, 1874) and a variety of Cingulata species (Galton, 1874; Marshall et al., 2021). This muscle has also been described in burrowing rodents such as *C*. *ludovicianus* (Scheetz et al., 2026), in carnivores such as *Felis catus* (domestic cat), *P*. *leo* (Galton, 1874) and *P*. *tigris* (Dunn et al., 2022) and may be present in humans (<30%) (Dellon, 1986; O'Driscoll et al., 1991). The variety of names (see Table 1) allocated to this muscle suggests that it originates in the proximity of the *Trochlea humeri*. In Pholidota, however, the muscle originated on the enlarged *Epicondylus medialis* (Steyn et al., 2018; present study) only, and the term *M. anconeus medialis* was therefore used for this muscle in *S*. *temminckii*.

### 4.2 Functional considerations

The thoracic limb muscles of Pholidota are involved in a variety of functions. These include locomotion, assistance with the adoption of a defensive posture, and digging for prey species.

#### 4.2.1 Locomotion

The thoracic limbs of Pholidota are either weight‐bearing in terrestrial species such as *Smutsia gigantea* (giant ground pangolin) and *M. pentadactyla* or assist in tree climbing in arboreal species such as *P*. *tetradactyla* and *P*. *tricuspis* (Pangolin Specialist Group, 2016). *S*. *temminckii*, however, presents a peculiarity in that it is terrestrial but bipedal, and therefore non‐weight‐bearing on its thoracic limbs. Thus, the thoracic limb muscles of *S*. *temminckii* differ from other terrestrial pangolin species and cursorial mammals in respect of their locomotory function.

The muscles of the thoracic limb can be divided into two groups: the proximal muscle group (extrinsic muscles), which attach the thoracic limb to the trunk, and the distal muscle group (intrinsic muscles), which are involved in locomotion and movement of the thoracic limb (Payne et al., 2005). The muscles of the proximal group are generally large, fusiform and display very short tendons or aponeurotic sheets for muscle attachment (Payne et al., 2005). The muscles of the distal group in weight‐bearing and cursorial mammals generally possess short muscle fibres and long tendons (Payne et al., 2005). The muscle bellies of the intrinsic muscles of the thoracic limb of *S*. *temminckii* were relatively well‐developed and possessed comparatively short tendons of insertion. This indicates that the thoracic limb muscles are not adapted to weight‐bearing during locomotion but rather adapted to developing force over a wide range of motions (Payne et al., 2005) (see below). To compensate for the non‐weight‐bearing thoracic limbs, *S*. *temminckii* possesses a broad, muscular tail (Swart, 2013) that assists with balance (Coombs, 1983). *S*. *temminckii* also displays a pelvis that is more vertically inclined than in other pangolin species, and this arrangement emphasises the greater use of the pelvic limbs for weight‐bearing (Kingdon, 1971; Swart, 2013).

The presence of a *Synsarcosis* (formation of a joint composed only of musculotendinous structures without bone articulation, as in the case of the extrinsic muscles attaching the thoracic limb to the trunk) (Kępa et al., 2023; Payne et al., 2004; Spataru et al., 2022) allows for a relatively free range of cranio‐caudal movements of the thoracic limb on the trunk (Payne et al., 2005). The extrinsic muscle group is involved in this movement (Payne et al., 2005) and leads to protraction and retraction of the thoracic limb during locomotion. The continuous, hard scale covering of Pholidota may, however, cause loss of mobility in the thoracic limbs and therefore impede locomotion (Kawashima et al., 2015). *M*. *pentadactyla* was observed to swing its head from side to side while walking, and this supports the concept of a walking style where opposed head swinging causes retraction of the *scapula* and *humerus* just before the swing phase (Kawashima et al., 2015). The well‐developed and extensive extrinsic musculature of the thoracic limb of Pholidota was therefore suggested to be an adaptation for the unique locomotor strategy employed, rather than for digging and climbing (Kawashima et al., 2015). The extrinsic musculature of *S*. *temminckii* is well‐developed (present study). The thoracic limbs are never fixed (as they would be during weight‐bearing in other Pholidota) and therefore the protractors of the thoracic limb that insert on the head, such as *M. brachiocephalicus*, *M. omocephalicus* and *M. omomastoideus*, would not be expected to have an effect on lateral movement of the head during locomotion. Although the extrinsic muscles of other Pholidota may be primarily involved in locomotion (Kawashima et al., 2015), the pronounced development of these muscles in *S*. *temminckii* points to functions other than locomotion (see below).

#### 4.2.2 Defence mechanism

Pholidota are peculiar in that they can adopt a defensive posture through ventral flexion to protect their vital organs when threatened (Heath, 1992a, 1992b). Two Cingulata species, *Tolypeutes tricinctus* (Brazilian three‐banded armadillo) and *Tolypeutes matacus* (southern three‐banded armadillo), also possess this ability when threatened by predators (Redford, 1994). A well‐developed *M. cutaneus trunci* (also termed *M. panniculus carnosis*) and fused *M. platysma* (cutaneous muscle of the face and neck) have been described in Cingulata species (Macalister, 1875; Marshall et al., 2021; Murie, 1872), and this muscle unit functions to protract the carapace and to assist the Cingulata with ventral flexion. In *S*. *temminckii*, *M. cutaneus trunci* displayed both an extensive site of origin on the trunk and insertion on the *Humerus*, respectively. The base of each scale was attached to the underlying *M. cutaneus trunci* by several thin tendons, and contraction of this muscle would assist in drawing the base of the scales towards the body. This action, linked to the overlapping nature of the scales (personal observation), may assist during ventral flexion of the pangolin's body. *M. cutaneus trunci* is extremely well‐developed and possesses extensive attachments to the trunk as well as to the thoracic limb in *M*. *pentadactyla* (Humphry, 1869; Kawashima et al., 2015). Similarly, in *S*. *temminckii*, this was the best‐developed muscle of the extrinsic group. Computed tomography of *S*. *temminckii* revealed that while in a defensive ball, the head was flexed towards the chest, and the thoracic limbs were protracted (Robert Kirberger, personal communication 2015). Protractors of the thoracic limb are therefore involved in the act of rolling up during the defensive posture of *S*. *temminckii*.

#### 4.2.3 Digging

*S*. *temminckii* uses its thoracic limbs for digging in hard soil surfaces and ant nests to find prey (Hoyt, 1987; Richer et al., 1997; Robinson, 2003), as well as to excavate previously occupied burrows (Heath, 1992b; Kingdon, 1971; Swart, 2013). The thoracic limbs would therefore have to perform a variety of functions, including protraction and retraction of the limbs, flexion and extension of the shoulder, elbow, carpal and digital joints, as well as pronation and supination of the antebrachium to assist in these tasks (Coombs, 1983; Hildebrand & Goslow, 2001b). In mammals, different forms of digging, such as chisel‐tooth digging, humeral‐rotation digging and digging employed by myrmecophagous mammals, such as hook‐and‐pull digging (Vermilingua) and scratch‐digging (Tubulidentata, Cingulata and Pholidota), have been identified (Hildebrand & Goslow, 2001b). Although Pholidota as a group were classified as scratch‐diggers (Hildebrand & Goslow, 2001b), this classification may not have specifically included *S*. *temminckii*.

Hook‐and‐pull digging employs well‐developed flexor and supinator muscles of the thoracic limb (Hildebrand & Goslow, 2001b). During this digging style, the animal hooks its claws into termite nests and soil and rips them open to secure prey species (Hildebrand & Goslow, 2001b). Scratch‐digging results from a sequence of flexion and extension of the joints of the thoracic limbs and is suitable for digging in hard substrates (Coombs, 1983; Hildebrand & Goslow, 2001b). The muscle mass of scratch‐digging mammals is concentrated in the retractors of the limb, extensors of the elbow joint and flexors of the carpus and digits (Olson et al., 2016; Warburton et al., 2013). Pronation and supination of the antebrachium is also reported (Coombs, 1983). Great force is required when digging in hard substrates and therefore scratch‐diggers display powerful muscles to generate the adequate out‐forces required (Hildebrand & Goslow, 2001b) (the term ‘out‐force’ is defined as the output force that is derived from the bone‐muscle system) (Hildebrand & Goslow, 2001a). One method of increasing the out‐force is by increasing the in‐force (Hildebrand & Goslow, 2001b) (the term ‘in‐force’ is defined as the input force applied to the bone‐muscle system) (Hildebrand & Goslow, 2001a). Extended sites of muscle attachment as well as enlarged muscle mass increase the in‐force and thereby increase the out‐forces needed for scratch‐digging (Hildebrand & Goslow, 2001b). A second method of increasing the out‐force is by increasing the related in‐lever of the muscles (Hildebrand & Goslow, 2001b) (the term ‘in‐lever’ is defined as the structure that transmits a force from the in‐force to the pivot) (Hildebrand & Goslow, 2001a). This is achieved by muscles attaching relatively far from the joints on which they act (Hildebrand & Goslow, 2001b). The thoracic limb muscles of *S*. *temminckii* displayed certain characteristics that demonstrate an adaptation to scratch‐digging and are elaborated on below.

Certain extrinsic muscles of the thoracic limb are well‐developed and possess extensive attachments in scratch‐digging mammals (Galton, 1869; Humphry, 1869; Olson et al., 2016; Peterka, 1936; Windle & Parsons, 1899). These muscles include *M. serratus ventralis* in a variety of Pholidota (Windle & Parsons, 1899) and Cingulata (Marshall et al., 2021; Olson et al., 2016; Windle & Parsons, 1899), *M. brachiocephalicus* as well as *M. cutaneus trunci* in Pholidota (Humphry, 1869; Windle & Parsons, 1899), *M. latissimus dorsi* in *C*. *ludovicianus* (Fain, 2021; Peterka, 1936; Rupert et al., 2015; Scheetz et al., 2026), *Euphractus sexcinctus* (six‐banded armadillo), *D*. *novemcinctus* (Windle & Parsons, 1899) and *Mm. pectorales superficiales* in *O*. *afer* (Galton, 1869) and *D*. *novemcinctus* (Olson et al., 2016). *S*. *temminckii* displayed a relatively well‐developed *M. brachiocephalicus, M. latissimus dorsi*, *Mm. pectorales superficiales*, and a massive *M. cutaneus trunci*. It is clear that the muscle mass of the extrinsic muscles of scratch‐digging mammals is concentrated in the retractors of the limb and therefore suggests that retraction of the limb is an important function during scratch‐digging (Olson et al., 2016; Warburton et al., 2013). However, the extrinsic musculature of *S*. *temminckii* is involved in its defence mechanism (protractors) (see above) as well as in scratch‐digging (retractors).

*M. coracobrachialis*, an extensor of the shoulder joint, is usually absent in scratch‐digging mammals such as *M*. *pentadactyla* (Humphry, 1869), and *Chlamyphorus truncatus* (pink fairy armadillo) (Windle & Parsons, 1899) but variably present in other Cingulata species (Marshall et al., 2021), a variety of burrowing rodents (Doubell et al., 2020; Fain, 2021; Sahd et al., 2023; Scheetz et al., 2026), and in the scratch‐digging marsupial, *I*. *obesulus* (Warburton et al., 2013). The muscle was also absent in *S*. *temminckii* (present study). The moderately sized *M. biceps brachii*, a flexor of the elbow joint, presented only one head in *M*. *pentadactyla* (Humphry, 1869; Windle & Parsons, 1899) and *S*. *temminckii* (present study). In *T*. *tetradactyla*, a hook‐and‐pull digger (Hildebrand & Goslow, 2001b), the above‐mentioned muscles were well‐developed. Additionally, *M. brachioradialis* and *M. extensor carpi radialis*, which can also act as flexors of the elbow joint, were also well‐developed in Vermilingua (Taylor, 1978). This suggests that shoulder extension and elbow flexion may be of less importance for the action of scratch‐digging than for hook‐and‐pull digging. Indeed, shoulder extension and elbow flexion are part of the recovery phase of scratch‐digging (as opposed to shoulder flexion and elbow extension during the power‐stroke in scratch‐digging), and the muscles associated with the recovery phase are generally smaller in mass (Martin et al., 2019).

*M. teres major* was described as typically well‐developed in the Pholidota, Cingulata, Vermilingua and Tubulidentata species (Windle & Parsons, 1899) as well as in *I*. *obesulus* (Warburton et al., 2013). This muscle is, however, not as well developed in *S*. *temminckii* (present study), which suggests that flexion of the shoulder joint may be less important in the function of the thoracic limb than in other pangolin and scratch‐digging species. Its moderate development may also be related to the extensive *M. triceps brachii Caput longum* (see above), which would largely incorporate the function of shoulder flexion over that of *M. teres major*. *S*. *temminckii* digs in more superficial substrates than other scratch‐digging mammals (Swart, 2013) and may also require less force during digging and thus less shoulder flexion.

The extensor muscles of the elbow joint are well‐developed in scratch‐digging mammals and certain muscles may even display additional bellies or extensive sites of origin (Galton, 1869; Humphry, 1869; Marshall et al., 2021; Olson et al., 2016; Warburton et al., 2013; Windle & Parsons, 1899). *M. triceps brachii* displays numerous scapular heads with additional sites of origin over the *Spina scapulae* in *M*. *pentadactyla* (Humphry, 1869) as well as in *M. javanica* and *P*. *tetradactyla* (Windle & Parsons, 1899). Likewise, *M. triceps brachii* displayed two (Galton, 1869) and sometimes three (Windle & Parsons, 1899) scapular heads in *O*. *afer* and two scapular heads in a variety of Cingulata species (Olson et al., 2016), as well as extensive sites of origin in *I*. *obesulus* and *M*. *lagotis* (Warburton et al., 2013). The scapular head of *M. triceps brachii* (*M. triceps brachii caput longum*) was very well‐developed in *S*. *temminckii* (present study) and displayed four individual bellies, named for their different origins on the *Scapula*. The caudal belly of the long head is comparable to the angular head described in certain Carnivora (Ercoli et al., 2015) and Cingulata (Marshall et al., 2021), but since it originates from the entire caudal margin of the *Scapula* (including, but not limited to, the caudal angle), the term ‘caudal belly’ was deemed more suitable. Although individual bellies are not named, the description of the extensive sites of origin of the long head is similar in *M*. *pentadactyla* (Humphry, 1869) and *S*. *temminckii* (present study). *M. triceps brachii* was the largest intrinsic muscle of the thoracic limb in this species. *M. anconeus medialis*, also an extensor muscle of the elbow joint, is typically present in all scratch‐digging myrmecophagous mammals (Galton, 1869; Humphry, 1869; Windle & Parsons, 1899) (see Table 1), previously known as the Edentata (see above), including *S*. *temminckii* (present study). Muscles that support an individual's body weight and maintain an upright posture function as extensors (Magnus, 1925) and are commonly referred to as ‘anti‐gravity’ muscles (Eng et al., 2008). In quadrupedal mammals, these muscles would include extensor muscles of the elbow joint (Armstrong et al., 1982), such as *M. triceps brachii* and *M. anconeus medialis*. Considering that *S*. *temminckii* does not bear weight on the thoracic limbs, the substantial development of *M. triceps brachii* and the presence of *M. anconeus medialis* indicate that extension of the elbow joint may be most important for scratch‐digging in this species.

Rotation of the antebrachium is an important function during digging (Coombs, 1983; Hildebrand & Goslow, 2001b) and is usually affected by *M. pronator teres*, *M. pronator quadratus*, *M. supinator* and *M. brachioradialis*. A well‐developed *M. pronator teres* that originates from the *Epicondylus medialis* and inserts on the distal *Radius*, with no attachment to the *Ulna*, is viewed as characteristic of the Edentata (Windle & Parsons, 1899). This well‐developed muscle, which was present in *S*. *temminckii* (present study), has been described in *P*. *tetradactyla* (Windle & Parsons, 1899), *P*. *tricuspis* (Windle & Parsons, 1899), *M*. *pentadactyla* (Humphry, 1869; Windle & Parsons, 1899) and *M. javanica* (Windle & Parsons, 1899), Cingulata (Marshall et al., 2021; Olson et al., 2016; Windle & Parsons, 1899) and *O*. *afer* (Endo et al., 2002; Galton, 1869; Humphry, 1869; Windle & Parsons, 1899). It has also been described in the scratch‐digging *I*. *obesulus* (Warburton et al., 2013), in scratch‐digging *Bathyergus* rodent species (Doubell et al., 2020; Montoya‐Sanhueza et al., 2024; Sahd et al., 2023) and *C*. *ludovicianus* (Scheetz et al., 2026), as well as in Vermilingua (Humphry, 1869; Taylor, 1978; Windle & Parsons, 1899), which are classified as hook‐and‐pull diggers (Hildebrand & Goslow, 2001b). *M. supinator* is well‐developed in scratch‐digging mammals such as *O*. *afer* (Galton, 1869; Windle & Parsons, 1899), *P*. *tricuspis* and *M. javanica* (Windle & Parsons, 1899), *M*. *pentadactyla* (Humphry, 1869; Windle & Parsons, 1899) and *S*. *temminckii* (present study) and in the hook‐and‐pull digging Vermilingua (Taylor, 1978; Windle & Parsons, 1899). However, the muscle is absent in Cingulata (Marshall et al., 2021; Olson et al., 2016; Windle & Parsons, 1899). Additionally, *M. brachioradialis* supinates the antebrachium and was especially well‐developed in certain Vermilingua (Taylor, 1978; Windle & Parsons, 1899) and in *O*. *afer* (Galton, 1869; Windle & Parsons, 1899). It was, however, absent in certain Cingulata (Galton, 1869; Marshall et al., 2021; Olson et al., 2016; Windle & Parsons, 1899) and in the Pholidota (Humphry, 1869; Windle & Parsons, 1899).

In contrast, *M. omoradialis* (also referred to as *M. supinator longus*), a potential supinator of the antebrachium, has only previously been described in pangolin species, for example, in *P*. *tricuspis*, *M. javanica* (Windle & Parsons, 1899) and *M*. *pentadactyla* (Humphry, 1869; Windle & Parsons, 1899) as well as in *S*. *temminckii* (present study). This muscle may perform a similar function to *M. brachioradialis* found in Vermilingua and Tubulidentata. *M. pronator quadratus* has not been reported in Pholidota (Humphry, 1869; Windle & Parsons, 1899, present study) or in (Galton, 1869; Windle & Parsons, 1899) and is only occasionally present in Cingulata (Marshall et al., 2021; Olson et al., 2016; Windle & Parsons, 1899). It is, however, well‐developed in Vermilingua (Humphry, 1869; Taylor, 1978). The absence of this muscle in the above‐mentioned scratch‐digging mammals suggests that, due to its presence in Vermilingua, together with the above‐mentioned muscles, it would appear that rotation of the antebrachium is of greater importance for hook‐and‐pull digging.

The superficial and deep flexor muscles of the digits displayed extensive development in certain scratch‐digging mammals (Humphry, 1869; Olson et al., 2016; Warburton et al., 2013; Windle & Parsons, 1899). *M. flexor digitorum superficialis* in pangolin species (Humphry, 1869; Windle & Parsons, 1899) demonstrated a large variability in the anatomical arrangement and site of origin for the different muscle bellies. In the terrestrial *M*. *pentadactyla* (Humphry, 1869; Windle & Parsons, 1899) and *M. javanica* (Windle & Parsons, 1899), *M. flexor digitorum superficialis* possessed five individual muscle bellies that each supplied a single digit only. In the arboreal *P*. *tetradactyla* and *P*. *tricuspis* (Windle & Parsons, 1899), as well as *S*. *temminckii* (present study), this muscle displayed four individual bellies, with one belly supplying a single digit only. The scratch‐digging *I*. *obesulus* and *M*. *lagotis* also displayed a well‐developed *M. flexor digitorum superficialis* (Warburton et al., 2013). The individual muscle bellies allow for greater fine control of the digits (Olson et al., 2016). The terrestrial *M*. *pentadactyla* and *M. javanica* possess more individual digital tendons of *M. flexor digitorum superficialis* than the arboreal *P*. *tetradactyla* and *P*. *tricuspis*, as well as the bipedal *S*. *temminckii*. Thus, *M. flexor digitorum superficialis* allows for finer control in digging, especially in terrestrial quadrupedal pangolin species when compared to arboreal species. *S*. *temminckii*, however, appears to be more aligned with arboreal pangolins in this respect.

*M. flexor digitorum profundus* is also greatly developed in scratch‐digging mammals such as *O*. *afer* (Galton, 1869; Windle & Parsons, 1899), Cingulata (Marshall et al., 2021; Olson et al., 2016; Windle & Parsons, 1899), *I*. *obesulus* (Warburton et al., 2013), *Marmota monax* (groundhog) (Rupert et al., 2015), *S. citellus* (Lagaria & Youlatos, 2006) and in *M*. *pentadactyla* (Humphry, 1869; Windle & Parsons, 1899), *P*. *tetradactyla* (Windle & Parsons, 1899) and *S*. *temminckii* (present study). Various heads have been described in these species, as well as variability in the number of muscle bellies. Although the nomenclature may differ between species and authors (N. A. V. terminology was used in the present study), it is clear that *M. flexor digitorum profundus* displays extensive areas of origin from the *Humerus*, *Ulna* and *Radius*. The muscle fibres merged over the palmar carpal area to form a broad tendon that was reported to contain a large, fibro‐cartilaginous sesamoid bone in a variety of Cingulata (Windle & Parsons, 1899; Olson et al., 2016; Marshall et al., 2021). A similar structure was also reportedly present in the *M. flexor digitorum profundus* tendon of *P*. *tetradactyla*, *M. javanica* (Windle & Parsons, 1899) and *M*. *pentadactyla* (Humphry, 1869; Windle & Parsons, 1899), but not as pronounced as in Cingulata (Windle & Parsons, 1899). *M. flexor digitorum profundus* of *O*. *afer* also formed a single broad tendon before dividing into digital tendons (Galton, 1869). However, this tendon did not possess a sesamoid bone as reported in Cingulata and Pholidota (Galton, 1869). *M. flexor digitorum profundus* provides unified movements of the digits in Cingulata, Pholidota and Tubulidentata, as all the muscle heads initially join into a single common tendon before giving rise to tendon slips to the individual digits (Olson et al., 2016). The presence of a sesamoid bone would increase the mechanical advantage of the muscle, allowing it to augment the total force applied to the substrate (Olson et al., 2016). A broad, fibrous carpal plate was observed in *S*. *temminckii* (present study). There was, however, no evidence of a fibro‐cartilaginous or osseous sesamoid bone. The absence of a sesamoid bone may be due to the relatively weaker force required while digging in superficial substrates (Swart, 2013) as compared to those scratch‐diggers mentioned above. *M. flexor digitorum profundus* provides a more powerful, unified digging action which is amplified in terrestrial quadrupedal pangolins that dig in deeper substrates as opposed to *S*. *temminckii*, which is not known to dig in deep substrates (Kingdon, 1971; Swart, 2013).

Movement of the metacarpophalangeal and digital joints in fossorial mammals is mostly limited to flexion and extension in the parasagittal plane, and little lateral movement occurs (Kley & Kearney, 2006). The presence of well‐developed muscles of the manus and individual digits in *S*. *temminckii*, such as *Mm. lumbricales*, *Mm*. *interossei* and *M. flexor digiti I brevis* (present study) is similar to what has been reported in other pangolin species (Humphry, 1869; Windle & Parsons, 1899) and suggests a greater degree of dexterity than would be expected. This situation is similar to the development of muscles of the manus and individual digits in carnivores as seen in *C. familiaris* (Evans & de Lahunta, 2013), *G*. *cuja* (Ercoli et al., 2015), *L. pardalis* (Julik et al., 2012), *A*. *fulgens* (Fisher et al., 2009), *P*. *tigris* (Dunn et al., 2022), and *Lynx lynx* (Eurasian lynx) (Viranta et al., 2016), rather than in scratch‐diggers such as Cingulata (Marshall et al., 2021; Olson et al., 2016; Windle & Parsons, 1899). Although the development of these muscles in Carnivora is possibly influenced by the requirement for holding on to prey, and for climbing in arboreal species (Viranta et al., 2016), the development of the individual digital muscles in pangolin Pholidota may be to aid in adducting the digits to form a shovel‐shaped manus which is employed during the power stroke of scratch‐digging as described in *T*. *taxus* (Moore et al., 2013).

## 5 CONCLUSIONS

The thoracic limb musculature of *S*. *temminckii* displays adaptations for the act of rolling up when threatened, non‐weight‐bearing locomotion and the ability for strong retraction of the limb, extension of the elbow joint, flexion of the digits, as well as moderate rotation of the antebrachium. The latter features support the classification of *S*. *temminckii* as a scratch‐digging mammal. The thoracic limb musculature also suggests the possibility of increased dexterity in the manus for an unknown reason, which needs to be investigated further. The findings of this study can provide valuable information when treating pangolins with musculoskeletal disorders, as well as support future comparative studies in both extinct and extant pangolin species.

## AUTHOR CONTRIBUTIONS

This work was conducted during the Master's degree study of Christine Steyn (C. S.) under the supervision of Martina Rachel Crole and John Thomson Soley. C. S. performed the dissections, wrote the initial draft and prepared the figures. All authors contributed to and approved the manuscript.

## CONFLICT OF INTEREST STATEMENT

There was no conflict of interest to report.

## Supporting information


**Table S1.** Extrinsic muscles of the thoracic limb.
**Table S2.** Muscles of the shoulder joint.
**Table S3.** Muscles of the elbow joint.
**Table S4.** Muscles of the radio‐ulnar joint.
**Table S5.** Muscles of the carpal and digital joints.
**Table S6.** Muscles of the forepaw and individual digits.
**Table S7.** List of species compared in the study, as well as their mode of digging, where applicable. Additional references to specific species are given in the text.
