# Supplementary material for: Thoracic limb myology of Smutsia temminckii (Temminck's pangolin)
Source: J Anat. 2026 Nov 14:10.1111/joa.70268. Online ahead of print. doi: 10.1111/joa.70268 (PMC13768876; doi:10.1111/joa.70268)
Supplement: Supplementary file 1 [file JOA-9999-0-s001.docx]

**Supplementary Material**

**Table S1: Extrinsic muscles of the thoracic limb**

| **Muscle** | **Origin** | **Insertion** | **Function** |
| --- | --- | --- | --- |
| ***M. trapezius*** (Figs. 1a,b; 6a) | | | |
| *Pars cervicalis* | Median raphe from C3-C7 and the contralateral muscle | Cranially on the *Spina scapulae* and *Acromion* | Protraction of the limb; elevation of the *Scapula* |
| *Pars thoracica* | *Lig. supraspinale* from T1-T10, the aponeurosis of the *M. serratus dorsalis caudalis* and the contralateral muscle | Caudally on the *Spina scapulae* | Retraction of the limb; elevation of the *Scapula* |
| ***M. omocephalicus**** (Figs. 1b; 6b) | | | |
| *Pars superficialis* | Cranial margin of the *Spina scapulae* | Dorso-medially on the *Crista nuchae* | Protraction of the limb |
| *Pars profunda* | Cranio-dorsal margin of the *Spina scapulae* | Midway on the *Crista nuchae* | Protraction of the limb |
| ***M. omo-***  ***mastoideus****  (Figs. 1b; 6b) | Cranio-ventral margin of the *Spina scapulae* | Ventro-laterally on the *Crista nuchae* and *Processus mastoideus* | Protraction of the limb |
| ***M. rhomboideus*** (Figs. 1a-c; 6a,b) | | | |
| *M. rhomboideus capitis* | *Angulus cranialis* and cranial insertion of the *M. rhomboideus cervicis* | Dorso-medially on the *Crista nuchae* | Protraction of the limb; elevation of the *Scapula* |
| *M. rhomboideus cervicis* | *Procc. spinosi* of C3-C7 and the contralateral muscle | Medially on the *Angulus cranialis*, *Margo dorsalis* and *Cartilago scapulae* | Protraction of the limb; elevation of the *Scapula* |
| *M. rhomboideus thoracis* | *Procc. spinosi* of T1-T7 and the contralateral muscle | Medially on the *Margo dorsalis*, *Angulus caudalis* and *Cartilago scapulae* | Retraction of the limb; elevation of the *Scapula* |
| ***M. serratus ventralis cervicis***  (Fig. 1b,c) | *Procc. transversi* of C3-C7 | Cranially on the *Facies serrata* | Protraction of the limb |
| ***M. serratus ventralis thoracis***  (Fig. 1c) | Mid-lateral surfaces of ribs 1-7 | Caudally on the *Facies serrata* | Retraction of the limb; muscle of inspiration |
| ***M. brachiocephalicus*** (Figs. 2a-c,6a) | | | |
| *M. cleido-*  *cephalicus* | *Intersectio clavicularis* | *Crista nuchae*, median raphe C1-C2 | Protraction of the limb |
| *M. cleido-*  *brachialis* | *Intersectio clavicularis* | *Crista humeri*, tendinous structure of the *Humerus* and the *Epicondylus medialis/lateralis* | Protraction of the limb |
| ***M. cutaneus trunci***  (Figs. 2a,c,d; 6a; 7a,b) | Dermis over lateral aspect of trunk | *Tuberculum minus*, *Crista tuberculi minoris, Tuberositas teres major*, tendinous structure of the *Humerus* and the *Epicondylus medialis* | Clamp scales down onto skin surface; retraction of the limb |
| ***M. latissimus dorsi***  (Figs. 2c;6a,b) | Lateral surfaces of ribs 7-11; dorsal thoracic aponeurosis | *Crista tuberculi minoris* and *Tuberositas teres major* | Retraction of the limb |
| ***Mm. pectorales superficiales*** (Figs. 2a-c; 7a,b) | | | |
| *M. pectoralis descendens* | Cranial part of *Corpus Sterni* and the contralateral muscle | Ventral part of *Crista humeri* | Adduction of the limb |
| *M. pectoralis transversus* | *Corpus Sterni*, base of *Proc. xiphoideus* and the contralateral muscle | Ventral part of *Crista humeri*; *Epicondylus medialis* | Adduction, protraction or retraction of the limb (depending on position of limb) |
| ***M. pectoralis profundus***  (Figs. 2a,c; 7b) | *Manubrium sterni* | Cranial part of *Tuberculum minus* | Adduction or protraction of the limb (depending on position of limb) |
| ***M. rectus sternalis****  (Fig. 7a,b) | Ventral margin of the *M. cutaneous trunci* | Ventral fascia and epimysium of the *M. obliquus externus abdominis* |  |

*Muscles that are not named in N.A.V.

**Table S2: Muscles of the shoulder joint**

| **Muscle** | **Origin** | **Insertion** | **Function on the shoulder joint** |
| --- | --- | --- | --- |
| ***M. supraspinatus***  (Figs. 1a; 2a,b; 8) | *Fossa supraspinatus* | *Tuberculum majus* | Extension; stabilization; tensing of the joint capsule |
| ***M. infraspinatus***  (Figs. 1a, 2b, 6) | *Fossa infraspinatus* | *Facies m. infraspinati* | Flexion; stabilization |
| ***M. omoradialis****  (Figs. 1a; 3; 6a; 8a,b; 9a) | Caudo-distal *Spina scapulae*, *Acromion* and aponeurosis covering the *M. infraspinatus* | Cranio-distal radius | Flexion; (also flexion of the elbow) |
| ***M. deltoideus***  (Figs. 1a,b; 2b,d; 8b) | Cranio-ventral *Acromion* | *Tuberositas deltoidea* | Flexion |
| ***M. teres major***  (Figs. 1c, 2c,d) | Medially on the *Margo caudalis* | *Crista tuberculi minoris* and *Tuberositas teres major* | Flexion; tensing of the joint capsule |
| ***M. teres minor***  (Figs. 1a, 2b) | *Tuberculum infraglenoidale* | *Tuberositas teres minor* | Flexion; tensing of the joint capsule |
| ***M. subscapularis***  (Figs. 1b,c, 2c,d) | *Fossa subscapularis* | *Tuberculum minus* | Stabilization; tensing of the joint capsule; extension, flexion or adduction (depending on the position of the limb) |

*Muscles that are not named in N.A.V.

**Table S3: Muscles of the elbow joint**

| **Muscle** | **Origin** | **Insertion** | **Function on the elbow joint** |
| --- | --- | --- | --- |
| ***M. triceps brachii*** (Figs. 1a,c, 2b-d, 4b-d, 6) | | | |
| *Caput longum*  *-Venter cranialis** | Caudo-distal *Spina scapulae* and *Acromion* | *Tuber olecrani*  (Fig. 3.4b,c,d) | Extension; (also flexion of the shoulder joint) |
| *-Venter medius** | Aponeurosis covering the *M. infraspinatus* |  |  |
| *-Venter caudalis** | Laterally on the *Margo caudalis* |  |  |
| *-Venter profundus** | *Tuberculum infraglenoidale* |  |  |
| *Caput lateralis* | *Linea m. tricipitis* | Proximo-laterally on the *Tuber olecrani* | Extension |
| *Caput medialis* | Caudo-medial humeral neck | Proximo-medially on the *Tuber olecrani* | Extension |
| *Caput accessorium* | Caudal humeral neck and joint capsule | Proximal part of *Tuber olecrani* | Extension; Tensing of the shoulder joint capsule |
| ***M. tensor fasciae antebrachii***  (Fig. 4d) | Ventral epimysium of the *M. latissimus dorsi* | Proximo-medially on the *Tuber olecrani* | Extension; tensing of the antebrachial fascia |
| ***M. anconeus***  (Figs. 2d, 4a) | *Epicondylus lateralis* and rim of the *Fossa olecrani* | Proximo-cranial *Olecranon* | Extension; tensing of the joint capsule |
| ***M. anconeus medialis****  (Figs. 2d, 4d, 8b) | *Epicondylus medialis* | Medial aspect of the *Tuber olecrani* | Extension |
| ***M. biceps brachii***  (Figs. 1, 4a,d, 7b,c) | *Tuberculum supraglenoidale* | Proximo-medially on the ulna | Flexion; (also extension of the shoulder joint |
| ***M. brachialis***  (Figs. 2b, 3, 4a,d, 7) | Proximo-laterally on the *Humerus* | Proximally on the cranio-medial ulna and *Tuberositas radii* | Flexion |

*Muscles that are not named in N.A.V.

**Table S4: Muscles of the radio-ulnar joint**

| **Muscle** | **Origin** | **Insertion** | **Function on the radio-ulnar joints** |
| --- | --- | --- | --- |
| ***M. pronator teres***  (Figs. 2a,c, 3a,b, 7) | Cranially on the *Epicondylus medialis* | Whole cranial margin of the radius | Pronation; (also flexion of the elbow joint) |
| ***M. supinator***  (Figs. 2a,b, 3a, 7c) | Distal *Epicondylus lateralis*, lateral collateral ligament of the elbow joint and the elbow joint capsule | Whole cranial margin of the radius | Supination; tensing of the elbow joint capsule; (also flexion of the elbow joint) |

**Table S5: Muscles of the carpal and digital joints**

| **Muscle** | **Origin** | | **Insertion** | | **Function on the carpal joint** |
| --- | --- | --- | --- | --- | --- |
| ***M. extensor carpi radialis***  (Figs. 2a,b, 5a, 7a,b) | Cranial surfaces of the *Epicondylus lateralis* and the *Crista supracondylaris lateralis* | | *Tuberositas ossis metacarpalis III* | | Extension; (also flexion of the elbow joint) |
| ***M. flexor carpi radialis***  (Figs. 2a, 5) | Cranio-laterally on the distal *Epicondylus medialis* | | Palmaro-medially on the palmar carpal fibrocartilage and proximo-medially on the *Os metacarpale I* | | Flexion; tensing of the carpal joint capsule |
| ***M. extensor carpi ulnaris*** (Figs. 2d, 4a,b, 5b, 7c) | | | | | |
| *Caput humerale* | Caudo-laterally on the distal *Epicondylus lateralis* | | Palmaro-laterally on the *Os metacarpale V* | | Flexion |
| *Caput ulnare* | Cranio-laterally on the *Olecranon* | | Palmaro-laterally on the *Os metacarpale V* | | Flexion |
| ***M. flexor carpi ulnaris***  (Figs. 4b,c, 5b, 8a) | Laterally on the *Olecranon* | | Proximal surface of the *Os carpi accessorium* | | Flexion |
| ***M. extensor digitorum communis***  (Figs. 2a,b, 5a, 7a,b) | Cranio-laterally on the *Epicondylus lateralis* | *Crista unguicularis* of the *Phalanges distales* of digits II-V | | Extension of the carpus and digits II-V | |
| ***M. extensor digitorum lateralis***  (Figs. 2a,b, 5, 7a,b) | Most cranio-lateral aspect of the *Epicondylus lateralis* | *Margo lateralis* of *Os metacarpale V* and the *M. extensor digitorum communis* tendon to digit IV | | Extension of the carpus and digits IV-V; abduction of digit V | |
| ***M. extensor digiti I et II***  (Figs. 3a, 4a,d, 5a, 7) | Cranially on the *Trochlea radii* and *Caput ulnae* | *Crista unguicularis* of the *Phalanges distales* of digits I-II | | Extension of the carpus and digits I-II | |
| ***M. abductor digiti I longus***  (Figs. 3a, 4a,d, 5, 7) | Cranially on the proximal *Corpus ulnae* and distally on the cranio-lateral *Corpus radii* | Proximally on the *Margo medialis* of *Os metacarpale I* (and *II*) | | Extension of the carpal joint; abduction of the carpometacarpal joint of digit I (and II) | |
| ***M. flexor digitorum superficialis*** (Fig. 2d, 4b,c,d, 5b, 8a) | | | | | |
| -*Venter digiti II** | Ventral epimysium of *M. anconeus medialis* and caudo-medially on the *Olecranon* | *Tuberositas flexoria* of the *Phalanx media* of digit II | | Flexion of the carpus and digit II | |
| -*Venter digiti III** | Caudally on the *Epicondylus medialis* and *Olecranon* | *Tuberositas flexoria* of the *Phalanx media* of digit III | | Flexion of the carpus and digit III | |
| -*Venter digiti IV** | Muscle fibres of *Venter digiti II* in mid-antebrachium | *Tuberositas flexoria* of the *Phalanx media* of digit IV | | Flexion of the carpus and digit IV | |
| -*Venter digiti V** | Muscle fibres of *Venter digiti IV* in metacarpal region | *Tuberositas flexoria* of the *Phalanx media* of digit V | | Flexion of digit V | |
| ***M. flexor digitorum profundus*** (Figs. 2a,c,d, 3c, 4b,c,d, 5b, 8b,c) | | | | | |
| *Caput humerale*  *-Venter medialis** | Cranio-medially on the distal *Epicondylus medialis* and  caudal aspect of the elbow joint capsule | *Tuberculum flexorium* of the *Phalanges distales* of digits I-V | | Flexion of the carpus and digits I-V; tensing of the elbow joint capsule | |
| *Caput humerale*  *-Venter lateralis** | Distally on the caudo-lateral *Epicondylus medialis* and the caudo-lateral aspect of the elbow joint capsule |  |  |  |  |
| *Caput ulnare*  *-Venter medialis** | Caudo-medially on the *Olecranon* and ulnar body and caudally on the elbow joint capsule |  |  |  |  |
| *Caput ulnare*  *-Venter lateralis** | Caudo-laterally on the Olecranon and ulnar body |  |  |  |  |
| *Caput radiale* | Caudo-laterally from the entire radial body and ventral part of the elbow joint capsule. |  |  |  |  |

*Muscles that are not named in the N.A.V.

**Table S6: Muscles of the forepaw and individual digits**

| **Muscle** | **Origin** | **Insertion** | **Function** |
| --- | --- | --- | --- |
| ***Mm. interflexorii***  (Fig. 5) | Disto-palmar aspect of the *M. flexor digitorum superficialis* over the proximal metacarpus | *Margo medialis* of *Os metacarpale II* and *Margo lateralis* of *Os metacarpale IV* | Flexion of metacarpophalan-  geal joints II and IV |
| ***M. flexor digitorum brevis*** | Lateral surface of the *M. flexor digitorum superficialis* in the distal carpus | Latero-palmarly on the metacarpophalangeal joint capsule of digit V | Flexion of metacarpophalan-  geal joint V |
| ***Mm. lumbricales***  (Figs. 5, 8a) | *M. flexor digitorum profundus* tendon in the distal carpus | Medially and laterally on the bodies of *Phalanges proximales* of digits III-V | Flexion of the metacarpophalan-  geal joints III-V |
| ***Mm. interossei***  (Fig. 5b) | Disto-palmarly on the carpal joint capsule and proximo-palmarly on *Ossa metacarpalia* *II-V* | Proximo-palmarly on *Phalanges proximales* of digits II-V | Flexion of the metacarpophalan-  geal joints II-V; tensing of the carpal joint capsule |
| ***M. abductor digiti I brevis***  (Fig. 5) | Medial projection of *Os carpale I* | Medial aspect of the metacarpophalangeal joint I | Abduction of digit I |
| ***M. flexor digiti I brevis***  (Figs. 5b, 8a) | Palmarly on *Os carpale I* | Disto-palmarly on *Os metacarpale I* | Flexion of digit I |
| ***M. adductor digiti I***  (Fig. 5b) | Caudo-laterally on *Os carpale I* | Disto-laterally on the *Os metacarpale I* | Adduction of digit I |
| ***M. abductor digiti V***  (Fig. 5) | Disto-lateral surface of *Os carpi accessorium* | *Margo lateralis* of *Os metacarpale V* | Abduction of digit V |
| ***M. flexor digiti V***  (Fig. 5b) | Caudo-laterally on *Os carpale IV* | *Facies palmaris* of *Os metacarpale V* | Flexion of digit V |

**Table S7: List of species compared in the study, as well as their mode of digging, where applicable. Additional references to specific species are given in the text.**

| **Species** | **Common name** | **Digging mode** | **Reference** |
| --- | --- | --- | --- |
| *Acinonyx jubatus* | Cheetah | N/A | Hudson *et al*., 2011 |
| *Ailurus fulgens* | Red panda | N/A | Fisher *et al*., 2009 |
| *Bathyergus janetta* | Namaqualand dune mole-rat | Scratch | Montoya-Sanhueza *et al*., 2024 |
| *Bathyergus suillus* | Cape dune mole-rat | Scratch | Doubell *et al.*, 2020 |
| *Bradypus tridactylus* | Pale-throated three-toed sloth | N/A | Humphry, 1869 |
| *Bradypus variegatus* | Brown-throated three-toed sloth | N/A | Olson *et al*., 2018 |
| *Canis familiaris* | Domestic dog | N/A | Williams *et al*., 2008 |
| *Chlamyphorus truncatus* | Pink fairy armadillo | Scratch | Windle & Parsons, 1899 |
| *Choloepus didactylus* | Linnaeus’s two-toed sloth | N/A | Humphry, 1869 |
| *Cyclopes didactylus* | Silky anteater | Hook-and-pull | Windle & Parsons, 1899 |
| *Cynomys ludovicianus* | Black-tailed prairie dog | Scratch | Scheetz *et al*., 2026 |
| *Dasypus novemcinctus* | Nine-banded armadillo | Scratch | Olson *et al.*, 2016 |
| *Euphractus sexcinctus* | Six-banded armadillo | Scratch | Windle & Parsons, 1899 |
| *Felis catus* | Domestic cat | N/A | Galton, 1874 |
| *Galictis cuja* | Lesser grison | N/A | Ercoli *et al*., 2015 |
| *Isoodon obesulus* | Southern brown bandicoot | Scratch | Warburton *et al*., 2013 |
| *Leopardus geoffroyi* | Geoffroy’s cat | N/A | Sánchez *et al*., 2019 |
| *Leopardus pardalis* | Ocelot | N/A | Julik *et al*., 2012 |
| *Leopardus wiedii* | Margay cat | N/A | da Costa da Silva *et al*., 2025 |
| *Lycalopex gymnocercus* | Pampas fox | N/A | de Souza Junior *et al*., 2018 |
| *Lynx lyns* | Eurasian lynx | N/A | Viranta et al., 2016 |
| *Macrotis lagotis* | Greater bilby | Scratch | Warburton *et al*., 2013 |
| *Manis javanica* | Malayan pangolin | Scratch | Windle & Parsons, 1899 |
| *Manis pentadactyla* | Chinese pangolin | Scratch | Humphry, 1869 |
| *Marmota monax* | Groundhog | Scratch | Rupert *et al*., 2015 |
| *Myrmecophaga tridactyla* | Giant anteater | Hook-and-pull | Windle & Parsons, 1899 |
| *Nasua nasua* | Ring-tailed coati | N/A | Tarquini *et al*., 2023 |
| *Orycteropus afer* | Aardvark | Scratch | Endo *et al*., 2002 |
| *Panthera leo* | Lion | N/A | Vargas *et al*., 2017 |
| *Panthera onca* | Jaguar | N/A | Sánchez *et al*., 2019 |
| *Panthera tigris* | Tiger | N/A | Dunn *et al*., 2022 |
| *Panthera uncia* | Snow leopard |  | Smith *et al*., 2021 |
| *Phataginus tetradactyla* | Long-tailed pangolin | Scratch | Windle & Parsons, 1899 |
| *Phataginus tricuspis* | White-bellied pangolin | Scratch | Windle & Parsons, 1899 |
| *Smutsia gigantea* | Giant ground pangolin | Scratch | Hildebrand & Goslow, 2001b |
| *Smutsia temminckii* | Temminck’s pangolin | Scratch | Present study |
| *Spermophilus citellus* | European ground squirrel | Scratch | Lagaria & Youlatos, 2006 |
| *Tamandua tetradactyla* | Lesser anteater | Hook-and-pull | Windle & Parsons, 1899 |
| *Taxidea taxus* | American badger | Scratch | Moore *et al*., 2013 |
| *Tolypeutes matacus* | Southern three-banded armadillo | Scratch | Redford, 1994 |
| *Tolypeutes tricinctus* | Brazilian three-banded armadillo | Scratch | Redford, 1994 |
